# Caspase-1/ASC Inflammasome-Mediated Activation of IL-1β–ROS–NF-κB Pathway for Control of *Trypanosoma cruzi* Replication and Survival Is Dispensable in NLRP3^−/−^ Macrophages

**DOI:** 10.1371/journal.pone.0111539

**Published:** 2014-11-05

**Authors:** Nilay Dey, Mala Sinha, Shivali Gupta, Mariela Natacha Gonzalez, Rong Fang, Janice J. Endsley, Bruce A. Luxon, Nisha Jain Garg

**Affiliations:** 1 Department of Microbiology and Immunology, University of Texas Medical Branch (UTMB), Galveston, Texas, United States of America; 2 Department of BioChemistry & Molecular Biology, UTMB, Galveston, Texas, United States of America; 3 Department of Pathology, UTMB, Galveston, Texas, United States of America; 4 Faculty of the Institute for Human Infections and Immunity and the Center for Tropical Diseases, UTMB, Galveston, Texas, United States of America; 5 Instituto Nacional de Parasitología “Dr. Mario Fatala Chaben”, Ciudad Autónoma de Buenos Aires, Argentina; Albert Einstein College of Medicine, United States of America

## Abstract

In this study, we have utilized wild-type (WT), ASC^−/−^, and NLRP3^−/−^ macrophages and inhibition approaches to investigate the mechanisms of inflammasome activation and their role in *Trypanosoma cruzi* infection. We also probed human macrophages and analyzed published microarray datasets from human fibroblasts, and endothelial and smooth muscle cells for *T. cruzi-*induced changes in the expression genes included in the RT Profiler Human Inflammasome arrays. *T. cruzi* infection elicited a subdued and delayed activation of inflammasome-related gene expression and IL-1β production in mφs in comparison to LPS-treated controls. When WT and ASC^−/−^ macrophages were treated with inhibitors of caspase-1, IL-1β, or NADPH oxidase, we found that IL-1β production by caspase-1/ASC inflammasome required reactive oxygen species (ROS) as a secondary signal. Moreover, IL-1β regulated NF-κB signaling of inflammatory cytokine gene expression and, subsequently, intracellular parasite replication in macrophages. NLRP3^−/−^ macrophages, despite an inability to elicit IL-1β activation and inflammatory cytokine gene expression, exhibited a 4-fold decline in intracellular parasites in comparison to that noted in matched WT controls. NLRP3^−/−^ macrophages were not refractory to *T. cruzi*, and instead exhibited a very high basal level of ROS (>100-fold higher than WT controls) that was maintained after infection in an IL-1β-independent manner and contributed to efficient parasite killing. We conclude that caspase-1/ASC inflammasomes play a significant role in the activation of IL-1β/ROS and NF-κB signaling of cytokine gene expression for *T. cruzi* control in human and mouse macrophages. However, NLRP3-mediated IL-1β/NFκB activation is dispensable and compensated for by ROS-mediated control of *T. cruzi* replication and survival in macrophages.

## Introduction

Chagas disease affects 11–18 million people world-wide [1]. Upon exposure to *Trypanosoma cruzi* (*T. cruzi* or *Tc*), infected individuals exhibit an acute phase of Chagas disease that lasts for a couple of months and is characterized by symptoms such as fever, fatigue, body aches, diarrhea, and vomiting. After the control of parasitemia, a majority of infected patients enter an indeterminate chronic phase that is marked by a lack of clinical symptoms of the acute phase. Ten to thirty years after initial infection, 30–40% of indeterminate phase patients progress to develop chagasic cardiomyopathy [2].

The studies in experimental models have shown that macrophages (mφs), as well as dendritic and natural killer cells, play an important role in control of *T. cruzi* infection [3]–[5]. The interaction of *T. cruzi* with mφs and other cell types involved in the innate immune response are mediated by pattern recognition receptors (PRRs) such as toll-like receptors (TLRs). Upon recognition of pathogen-assoCiated molecular patterns (PAMPs), TLRs transmit the signal via cytoplasmic domains for the recruitment of cytosolic adaptor molecules, including myeloid differentiation primary-response protein 88 (MyD88), and subsequently induce nuclear factor κB (NFκB) activation, leading to the production of inflammatory cytokines and linking an innate response to an adaptive immune response (reviewed in [4]). *T. cruzi-*derived glycosylphosphatidylinositols and mucins have been shown to serve as PAMPs in engaging TLR signaling of a cytokine response. Others have demonstrated that TLR4 and TLR9 are engaged by parasite-derived glycosylinositol phospholipids and DNA, respectively, during the activation of host innate immune response leading to regulation of infection [6], [7]. *T. cruzi* also expresses cruzipain, a kinin-releasing cysteine protease, which induces dendritic cells maturation via activation of bradykinin (BK) B_2_ receptors (B_2_R) [8], [9].

A newly discovered family of PRRs is named Nucleotide-binding oligomerization domain (NOD) like receptors (NLRs) [10], [11]. NLRs have a tripartite domain structure and are characterized by the presence of a central nucleotide-binding oligomerization domain (NOD), also called NACHT domain, present in neuronal apoptosis inhibitor proteins (NAIP) and a C-terminal leucine-rich repeats (LRRs) domain of variable length (20–29 amino acids). The N-terminal effector binding region consists of a protein-to-protein interaction domain, i.e., Pyrin domain (PYD), a caspase recruitment domain (CARD), or baculovirus inhibitor of an apoptosis protein repeat (BIR) domain. Based upon the presence of PYD, CARD and BIR effector domains, NLRs are classified as NLRP, NLRC, and NAIP, respectively [11], [12]. Currently known members of the NLR family in humans include seven NLRCs (NLRC1-NLRC5, NLRX, and CIITA or NLRA), fourteen NLRPs (NLRP1-NLRP14), and seven NAIPs (NAIP1-NAIP7). The multi-meric protein macromolecules formed by NLRs are named inflammasomes. The most studied NLRP1 and NLRP3 inflammasomes recruit ASC (apoptosis-assoCiated, speck-like protein containing a CARD domain) and caspase-1 proteins. The ASC-dependent cleavage and activation of caspase-1 results in the formation of an active complex responsible for converting to active forms of pro-IL-1β (31 kDa to 17 kDa) and pro-IL-18 (24 kDa to 18 kDa) [13] and the activation of the inflammatory cytokine response.

In the context of pathogens invading the heart, it is recognized that besides innate immune cells, both endothelial and vascular smooth muscle cells (VSMCs) can also sense and respond to pathogens (or PAMPs) [14]–[16]. CardiomyoCytes, the main type of cells in the heart, and heart resident fibroblasts also express TLRs and/or NLRs [17], [18].

In this study, we have utilized wild type (WT), ASC^−/−^ and NLRP3^−/−^ mφs and inhibitory approaches to investigate the mechanisms of inflammasome activation and their role in the context of *T. cruzi* infection. We also probed the RT Profiler PCR Array System to identify the inflammasome-related changes induced by *T. cruzi* infection of human mφs and analyzed the published microarray datasets from *T. cruzi*-infected fibroblasts, and smooth muscle and endothelial cells for the change in expression of the 84 genes included in the inflammasome arrays. Our data demonstrate that *T. cruzi* infection, in comparison to treatment with LPS, elicits a subdued activation of inflammatory gene expression and IL-1β production in mφs. Yet, caspase-1/ASC inflammasome-dependent activation of the IL-1β – reactive oxygen species (ROS) – NF-κB pathway played an important role in control of *T. cruzi* replication in mφs. Further, NLRP3 controlled the ROS levels in mφs, and NLRP3 deficiency resulted in a potent increase in ROS-mediated parasite killing in infected mφs. To the best of our knowledge, this is the first study demonstrating a double-edged role of NLRP3 in determining mφ activation of ROS and cytokine response, both of which are required for clearance of *T. cruzi* infection.

## Materials and Methods

### Ethics statement

All animal experiments were performed according to the National Institutes of Health Guide for Care and Use of Experimental Animals and approved by the UTMB's Animal Care and Use Committee (protoCol # 08-05-029).

### Mice, parasites, and cells

C57BL/6 female mice (6–8-weeks old) were purchased from Harlan Labs (Indianapolis, IN). NLRP3^−/−^ and ASC^−/−^ mice (C57BL/6 background) were a gift from Dr V. Dixit (Genentech, San Francisco, CA) and bred at the UTMB animal facility. *T. cruzi* (SylvioX10/4 strain) trypomastigotes were maintained and propagated by the continuous *in vitro* passage of parasites in monolayers of C2C12 cells (an immortalized mouse myoblast cell line). *T. cruzi* isolate and C2C12 cells were purchased from American Type Culture Collection (ATCC, Manassas VA).

Single-cell suspensions of bone marrow (BM) - derived monoCytes from WT, ASC^−/−^ and NLRP3^−/−^ mice (C57BL/6 background) were added to petri dishes (10^6^ cells/ml) in complete RPMI media containing 20 ng/ml murine macrophage–colony stimulating factor (M-CSF, eBioscience, San Diego, CA) and incubated at 37°C in 5% CO_2_ for 10 days to support differentiation to mφs. The differentiated BM mφs were maintained in the presence of 5 ng/ml M-CSF during experimental use. THP-1 human monoCytes were differentiated into mφs by overnight incubation with 50 ng/ml phorbol-12-myristate-13-acetate (PMA), and then rested at 37°C/5% CO_2_ for 48 h in RPMI complete media containing 10% FBS. RAW 264.7 murine mφs were routinely cultured in DMEM with 10% FBS.

In general, primary or cultured mφs (0.5−1×10^6^ cells/ml) were seeded in Nunc Lab-Tek II chamber slides or 24-well or 6-well plates, infected with *Tc* trypomastigotes (cell: parasite ratio, 1∶3) for 2 h, washed to remove free parasites, and then incubated for 3, 6, 12, and 18 h. In some experiments, 5 mM ATP was added during the last 30 min of incubation. When monitoring the role of inflammasomes or NADPH oxidase (NOX2)-mediated ROS in parasite control, infected mφs were incubated in the presence of 1 µg/ml anti-IL-1β antibody (Santa Cruz, Dallas TX); 20 µM Ac-YVAD-CHO (caspase-1 inhibitor, Enzo Life Sc., Farmingdale, NY); 100 ng/ml cycloheximide (inhibits protein biosynthesis); 50 µM glibenclamide (bloCks the maturation of caspase-1 and pro-IL-1β by inhibiting K^+^ efflux and also inhibits NLRP3 inflammasome activation (Imgenex, San Diego, CA); 10 µM diphenylene iodonium (DPI) or 30 µM apoCynin (inhibitors of NOX2/ROS); and 1 mM N-acetylcysteine (NAC, ROS scavenger). Macrophages incubated with media alone or LPS (100 ng/ml) were used as controls. Cells and culture supernatants were stored at −80°C.

### Probing the RT Profiler Human Inflammasome PCR Arrays

THP-1 cells, with and without *T. cruzi* infection and treatments, were harvested with 500 µl Bio-Rad cell lysis/RNA extraction buffer. Total RNA was extracted by using an Aurum DNA-free RNA isolation kit (Bio-Rad, Hercules, CA) and measured at 260 and 280 nm for determination of purity and concentration. The cDNA probes were generated by reverse transcription of 5 µg total RNA by using the Bio-Rad iScript cDNA synthesis kit.

The 96-well RT Profiler Human Inflammasome PCR Arrays (SA Biosciences/Qiagen, Valencia, CA) containing primer pairs for 84 key genes involved in the function of inflammasomes and NLR signaling were probed with 2 µl of cDNA template in the presence of 6.5 µl of dNTPs, MgCl_2_, and stabilizers (iQ SYBR Supermix, Bio-Rad), and PCR was carried out on a LightCycler 480 Multiple Plate System. A total of 42 arrays were probed with 14 research samples in triplicate, and datasets were analyzed by Web-based PCR Array Data Analysis software (SA Biosciences) for threshold cycle (Ct) value determination.

The Ct values from qPCR data were analyzed by using the open source HTqPCR v.1.12 software package [19]. Briefly, all array data were imported into HTqPCR, normalized by the quantile method and then filtered to exclude genes that exhibited Ct values >35. The relative expression level of each target gene in infected cells was calculated by using the formula, fold change  = 2^−ΔΔCt^, where ΔC_t_ represents the C_t_ (*Tc*-infected or LPS-treated sample) - C_t_ (control). The LimmaCt routine in the HT-qPCR package was employed for contrast analysis of all groups included in the experiment and for the identification of genes that were overall differentially expressed. LimmaCt utilizes the Linear Model for Microarray data (limma) R package to fit linear models for analyzing designed experiments and for the assessment of differential expressions in microarray data to perform contrast analysis between the different experimental groups. LimmaCt uses Empirical Bayesian methods from the eBayes function in limma to provide stable results even when the number of arrays is small. The basic statistics used for significance analysis consists of moderated t-statistics with the same interpretation as ordinary t-statistics computed for each gene and contrasts, except that the standard errors are shrunk towards a common value by using a Bayesian model. The eBayes function computes moderated F-statistics which combines all of the t-statistics from all the contrasts to calculate the significance of a gene(s). This F-statistic determines if gene(s) are differentially expressed across any contrast. The p-value is calculated based on the moderated t-statistics and F-statistics followed by an FDR adjustment. LimmaCt with the top table module was used to gain an appreciation of the change in differential expression of a particular gene over time (or treatment), and significance in changes in gene expression was accepted at p<0.05.

In some experiments, quantitative RT-PCR was performed for IL-1β, IL-18, CXCL1, and TNF-α mRNA levels and house-keeping genes (GAPDH and β-actin) with gene-specific primer pairs (Table S1) and iQ SYBR Supermix on a C1000 Touch Thermal Cycler (Bio-Rad). The relative expression level of each target gene was calculated by using the formula, fold change  = 2^−ΔΔCt^, where ΔC_t_ represents the C_t_ (infected sample) - C_t_ (control).

### Functional analysis

Datasets for differential expression of the genes included in RT Profiler Human Inflammasome PCR Arrays in human foreskin fibroblasts (HFF), human microvascular endothelial cells (HMVEC), and human vascular smooth muscle cells (HVSMC), infected with *T. cruzi* for 24 h, were obtained from the HG_U133 plus 2.0 Affymetrix array analysis data posted at Gene Expression Omnibus [20]. The selected differentially expressed gene datasets from *Tc*-infected HFF, HMVEC, and VSMC cells were submitted to Ingenuity iReport Analysis (Ingenuity Systems, Redwood city, CA). The iReport retrieves a set of biological information such as gene name, sub-cellular loCation, tissue specificity, function, assoCiation with disease, and integrates into networks and signaling pathways with biological meaning and significance. An e-value was calculated by estimating the probability of a random set of genes having a frequency of annotation for that term greater than the frequency obtained in the real set, and a threshold of e value <10^−3^ was set to retrieve significant molecular functions and biological processes.

### Parasite infectivity and replication in mφs

*T. cruzi* trypomastigotes were labeled with 5 µM SYTO^®^11 (binds DNA, Molecular Probes-Invitrogen, Eugene, OR) or 5 µM carboxyfluorescein succinimidyl ester (CFSE, binds amines, Invitrogen) for 20 min at 37oC. THP-1- or BM- derived mφs were infected and incubated with labeled *T. cruzi* trypomastigotes, as above. Cells were washed, and SYTO^®^11 or CFSE fluorescence as an indicator of parasite uptake was determined by using an Olympus BX-15 microscope equipped with a digital camera (magnification 40X). Cells infected with CFSE-labeled parasites were also fixed with 2% paraformaldehyde and visualized on a FACSCalibur flow cytometer (BD Biosciences, San Jose, CA) acquiring 20,000 events. Further analysis was performed by using FlowJo software (ver. 7.6.5, Tree-Star, San Carlo, CA). Mean Fluorescence intensity (MFI) of CSFE positive cells was used as a relative marker of parasites per cell.

Total DNA from normal and infected cells was isolated by using TRIzol reagent (Life Technologies, Grand Island, NY). Total DNA (100 ng) was used as a template in a quantitative PCR (qPCR) on an iCycler thermal cycler with SYBR Green Supermix (Bio-Rad) and oligonucleotide pairs specific for *Tc*18S ribosomal DNA (Table S1). Data were normalized to murine or human GAPDH, and fold change calculated as 2^−ΔΔCt^, where ΔΔC_t_ represents the C_t_ (sample) - C_t_ (control).

### Activation of mφs by *T. cruzi*: ROS, nitric oxide (^•^NO) and cytokines levels

Mφs were infected with *T. cruzi* for 1 h, washed to remove free parasites, and then incubated for up to 18 h, as above. Cells were incubated with 5 µM Carboxymethyl-2′,7′-dichlorodihydrofluorescein diacetate (CM-H_2_DCF-DA, from Life Technologies, Ex_495_/Em_527_, fluoresces green upon oxidation by intracellular ROS) for 30 min, and the fluorescence was recorded using a SpectraMax M5 microplate reader. In some experiments, cells were incubated for 30 min with 5 µM dihydroethidium. (DHE, Ex_518nm_/Em_605nm_, fluoresces red upon oxidation and binds DNA). Micrographs of DCF or DHE fluorescence were visualized on an Olympus BX-15 microscope, and images were captured by using a mounted digital camera (magnification 40X).

ROS release in supernatants was determined by an Amplex red assay. Briefly, 50 µl of supernatants from infected mφs were added in triplicate to 96-well, flat-bottomed plates, and mixed with a similar volume of 100 µM 10-acetyl 3, 7-dihydroxyphenoxazine (Amplex Red, Life Technologies) and 0.3 U/ml horseradish peroxidase. The H_2_O_2_-dependent oxidation of Amplex red to red fluorescent resorufin (Ex_563nm_/Em_587nm_) was recorded as above (standard curve: 50 nM - 5 µM H_2_O_2_) [21].

The ^•^NO level (indicator of iNOS activity) was monitored by the Greiss reagent assay and by using the Nitrate/Nitrite Colorimetric Assay Kit (Cayman, Ann Arbor, MI). Briefly, culture supernatants (50 µl) were reduced with 0.01 unit/100 µl of nitrite reductase, and incubated for 10 min with 100 µl of 1% sulfanilamide prepared in 5% phosphoric acid/0.1% N-(1-napthyl) ethylenediamine dihydroChloride (1∶1, v/v). After incubation for 10 min, formation of diazonium salt was monitored at 545 nm (standard curve: 2–50 µM sodium nitrite).

Culture supernatants from mφs incubated with *T. cruzi* (± inhibitors) were also utilized for measuring IL-1β and IL-18 release by using OptEIA ELISA kits (eBioscience), according to the manufacturer's instructions.

### Transient transfection and luciferase assay

RAW 264.7 cells were plated in 6-well plates, and, when at >70% confluency, transfected with pGL4.NF-κB-Luc reporter plasmid (3 µg/well, Promega, San Diego, CA) by using JetPEI transfection reagent (Polyplus transfection, New York, NY), according to instructions provided by the manufacturer. The pREP7-Rluc plasmid (500 ng) expressing renilla luciferase was co-transfected into RAW macrophages and used as an internal control reporter. After 30 h of transfection, cells were washed, replenished with complete medium, and infected with *T. cruzi* (± inflammasome inhibitors) for 18 h (positive control: 10 ng/ml recombinant TNF-α for 6 h). The relative NF-κB transcriptional activity was detected by using a Steady-Glo luciferase assay system (Promega) and recorded on a luminometer (Turner Biosystems, Sunnyvale, CA).

### Statistical Analysis

All experiments were conducted at least twice with triplicate observations per sample per experiment. All data were analyzed by using Graph Pad InStat ver.3 software and expressed as mean ± SD. Data were analyzed by the Student's *t* test (comparison of 2 groups) and 1-way analysis of variance (ANOVA) with Tukey's post-hoC test (comparison of multiple groups). Significance is shown by *^,#^ *p*<0.05, **^,##^ *p*<0.01, ***^,###^ *p*<0.001 (*normal-versus-infected; ^#^infected versus infected/treated).

## Results

### Mφs elicit subdued IL-1β response to *T. cruzi* infection

**Figure 1:**
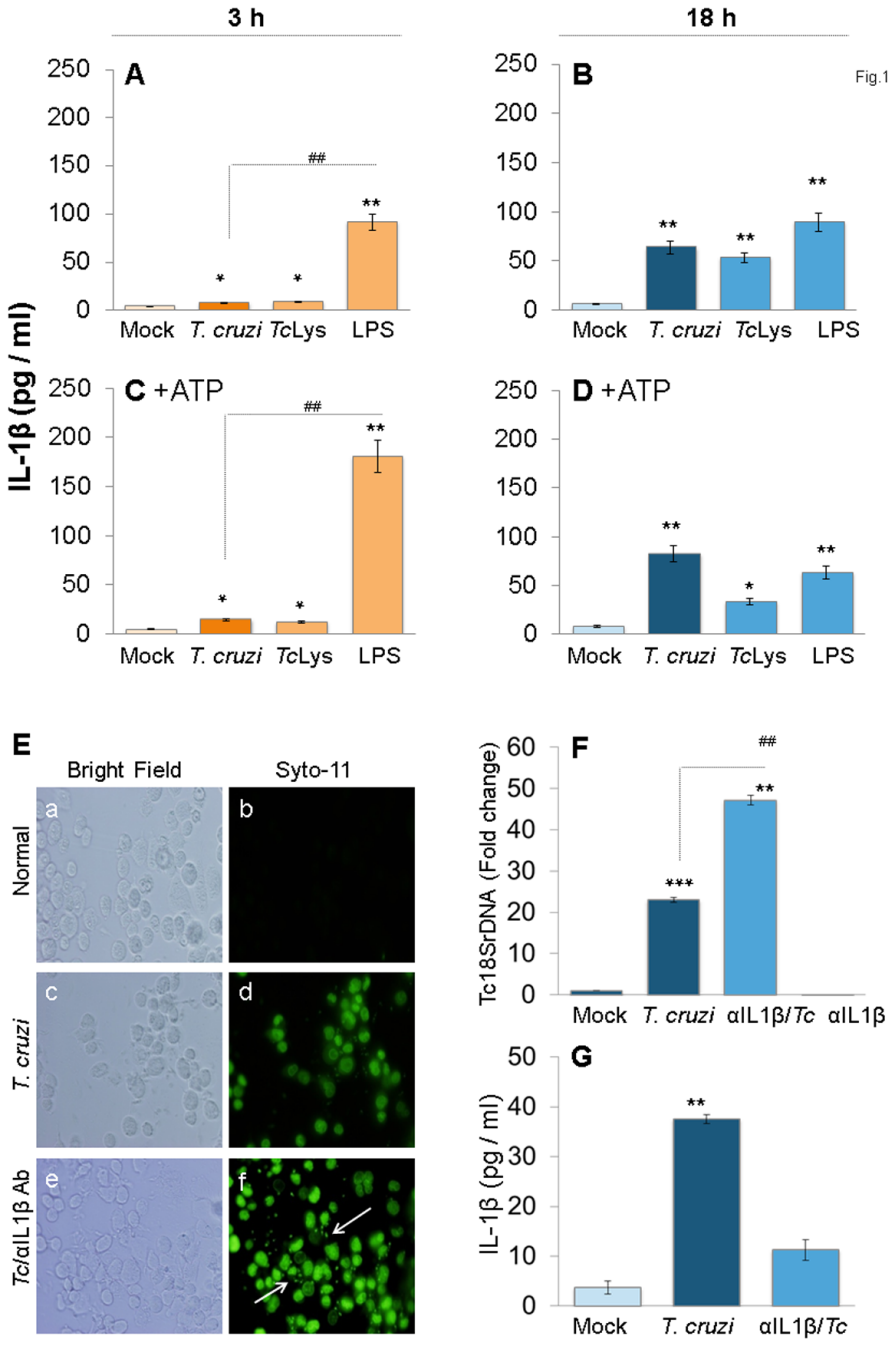
IL-1β production in macrophages infected by *T. cruzi*. (A–D) PMA-differentiated THP-1 mφs were incubated with *T. cruzi* trypomastigotes (cell: parasite ratio, 1∶3), *Tc* lysate (10 µg protein/10^6^ cells) or LPS (100 ng/ml) for 3 h *(A&C)* and 18 h *(B&D)*. In some experiments, ATP was added during last 30 min of incubation *(C&D)*. IL-1β release in supernatants was determined by ELISA. (E–G) IL-1β contributes to parasite control in mφs. THP-1 mφs were incubated with SYTO^®^11-labeled *T. cruzi* in the presence or absence of anti-IL-1β antibody for 18 h. *(E)* SYTO^®^11 fluorescence as an indicator of parasite uptake (shown by arrows) was determined by using an Olympus BX-15 microscope equipped with a digital camera (magnification 40X). *(F)* Quantitative PCR analysis of parasite burden in infected mφs by using *Tc18SrDNA*-specific oligonucleotides (normalized to human *GAPDH*). *(G)* Addition of anti-IL-1β antibody depletes secreted IL-1β levels in *T. cruzi-*infected mφs. In all figures, data are representative of three independent experiments and presented as mean ± SD. Significance is shown by *normal versus infected and ^#^treated/infected versus infected (*^,#^p<0.05, **^,##^p<0.01, and ***^,###^p<0.001).

THP-1 mφs incubated with *T. cruzi* trypomastigotes (1∶3, cell: parasite ratio) or *Tc*-lysate exhibited a ∼2-fold increase in IL-1β release at 3 h pi that was consistently increased at 6 h and 12 h pi (data not shown) and maximized to a>9.8-fold increase by 18 h pi (Fig.1A&B). Incubation with higher number of parasites (1∶4, 1∶5 or 1∶6, cell: parasite ratio) did not result in a further increase in IL-1β release at 3 h and 18 h pi (data not shown). LPS treatment (100-ng/ml) triggered a substantially higher level of IL-1β release in THP-1 mφs than was observed with *T. cruzi* infection, the maximal difference being noted at 3 h (Fig.1A&B). Exogenous addition of ATP, the K^+^ flux agent that can trigger caspase-1 cleavage and inflammasome activation in response to PAMPs, elicited a 2-fold and no increase in IL-1β release in *Tc-*infected and LPS-treated cells at 3 h (Fig.1A&C). No significant effect of exogenous ATP on IL-1β release was observed at 18 h post-incubation (Fig.1B&D)

To determine if IL-1β is required for parasite control, mφs were infected with SYTO^®^11-labeled *T. cruzi* and incubated in the presence or absence of anti-IL-1β antibody for 18 h (Fig.1E). The intracellular SYTO^®^11 fluorescence (indicates parasite presence) was significantly increased in anti-IL-1β antibody - treated THP-1 mφs (Fig.1E.d&f). The qPCR estimation of parasite burden confirmed the microscopic findings and showed a 2-fold increase in *Tc*18SrDNA signal in infected cells treated with anti-IL-1β antibody (Fig.1F). Antibody efficacy in depleting secreted IL-1β is shown in Fig.1G. Together, these data suggested that *a)* mφs respond to *T. cruzi* infection, in comparison to LPS treatment, by a subdued IL-1β release; b) IL-1β release can be enhanced by ATP at an early time-point pi; and c) IL-1β is required for controlling intracellular *T. cruzi*.

### Inflammasome-related gene expression in mφs infected by *T. cruzi*

**Figure 2:**
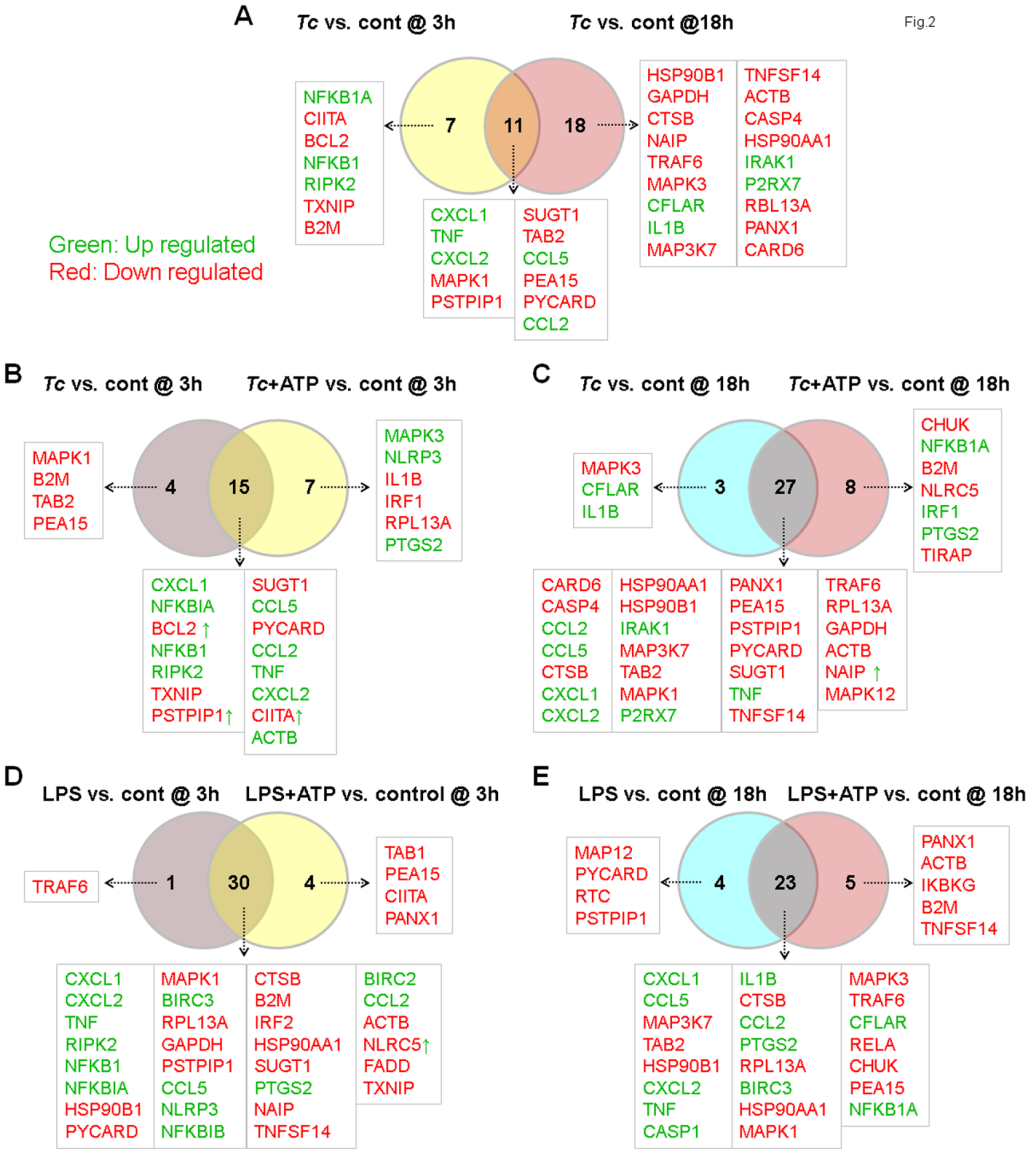
Venn diagram of inflammasome-related differential gene expression in mφs infected with *T. cruzi* (±ATP). THP-1 mφs were incubated with *T. cruzi* or LPS (± ATP treatment) as in Fig. 1. Total RNA was isolated, and cDNA was used as a template to probe the expression of 95 genes (including house-keeping genes) in the RT^2^ Profiler Inflammasome PCR Arrays. The differential mRNA level was captured by quantitative RT-PCR, normalized to housekeeping genes, and HTqPCR was employed to attain the statistically significant differential expression in treated- versus-control samples (Table 1 and Table S2). Shown are Venn diagrams of comparative analysis of gene expression in *T. cruzi*-infected mφs at 3 h versus 18 h *(A)*, effect of ATP stimulus on gene expression at 3 h *(B)* and 18 h *(C)* pi, and comparative effect of ATP stimulus on gene expression in LPS-treated mφs at 3 h *(D)* and 18 h *(E)*. Differential up-regulation (green) and down-regulation (red) of genes with respect to controls is presented. Genes presenting as red with green arrow in *B*–*F* showed decreased expression without ATP but were up-regulated by ATP treatment (and vice versa).

We investigated the mRNA expression levels of various genes involved in the function of inflammasomes and NOD-like receptor (NLR) signaling. THP-1 mφs were incubated with *T. cruzi* or LPS for 3 h and 18 h (± ATP treatment) and probed for the expression of 95 genes (including house-keeping genes) utilizing the RT^2^ Profiler Inflammasome PCR Arrays. The differential mRNA level was captured by qRT-PCR, and HTqPCR software was employed to attain the statistically significant differential expression in *Tc-*infected versus control mφs (± ATP) and LPS-treated versus control mφs at 3 h and 18 h (Table 1 & Table S2). Venn diagrams of comparative analysis of gene expression in *Tc*-infected and LPS-treated mφs at 3 h and 18 h (± ATP) are shown in Fig.2. Of the 95 genes that were examined, 63 genes exhibited differential expression in one of the studied groups (p<0.05). We noted the differential expression of 18 (8 up-regulated and 10 down-regulated) and 29 (9 up-regulated and 20 down-regulated) genes in *Tc*-infected mφs at 3 h and 18 h, respectively, in comparison to that noted in controls (Fig.2A, Table 1). When *Tc*-infected mφs were treated with exogenous ATP during the last 30 min of incubation, 22 (15 up-regulated and 7 down-regulated) and 35 (11 up-regulated and 24 down-regulated) of the inflammasome-related genes were differentially expressed at 3 h and 18 h pi, respectively (Fig. 2B & C, Table 1). Only seven and eight genes were uniquely expressed in *Tc*/ATP-treated mφs when compared to *Tc*-infected mφs at 3 h and 18 h, respectively. However, other genes, e.g., BCL2, PSTPIP1, and CIITA at 3 h and NAIP1 at 18 h, that were down-regulated by *T. cruzi* infection, were up regulated when ATP was provided exogenously (Fig.2B&C, Table 1). In LPS-treated mφs, we noted differential expression of 31 (12 up-regulated and 19 down-regulated) and 27 (11 up-regulated and 16 down-regulated) of the inflammasome-related genes at 3 h and 18 h pi, respectively, and the LPS-induced gene expression profile was not changed by exogenous addition of ATP (Fig.2D&E, Table S2). These data suggest that very few of the inflammasome-related genes are up regulated in response to *T. cruzi* infection. Exogenous ATP was effective in enhancing the inflammasome-related gene expression at 3 h, but not at 18 h pi. In comparison, LPS served as a potent activator, as evidenced by a significant up regulation of inflammasome-related gene expression within 3 h post-treatment. These data support the results presented in Fig.1 and suggest that *T. cruzi* is a silent invader that elicits low level of inflammatory response in macrophages.

**Table 1:** Inflammasome-related differential gene expression in THP-1 macrophages in response to *T. cruzi* infection (± ATP) in comparison to normal controls.

*Tc* vs control at 3h			*Tc* + ATP vs control at 3 h			*Tc* + ATP vs *Tc* at 3 h		
Gene name	ddCt log ratio	p value	Gene name	ddCt log ratio	p value	Gene name	ddCt log ratio	p value
CXCL1	−4.10	0.000	CXCL1	−4.48	0.000	ACTB	−2.41	0.002
TNF	−2.86	0.000	TNF	−3.68	0.000	CXCL2	−2.21	0.001
NFKBIA	−2.46	0.000	CXCL2	−5.36	0.000	PTGS2	−4.73	0.020
CXCL2	−3.14	0.000	NFKBIA	−3.24	0.000	TNF	−0.82	0.028
CIITA	3.07	0.001	TXNIP	2.46	0.000	CIITA	−1.68	0.037
BCL2	0.95	0.005	ACTB	−2.79	0.001	NLRP3	−0.65	0.051
NFKB1	−1.03	0.007	RIPK2	−1.99	0.001			
RIPK2	−1.43	0.008	NFKB1	−1.25	0.002			
TXNIP	1.56	0.007	MAPK3	1.42	0.006			
MAPK1	0.79	0.010	PSTPIP1	1.11	0.006			
PSTPIP1	1.03	0.010	NLRP3	−0.96	0.007			
B2M	1.44	0.023	BCL2	0.86	0.009			
SUGT1	0.86	0.026	SUGT1	1.03	0.010			
TAB2	0.66	0.032	PYCARD	1.10	0.015			
CCL5	−1.12	0.042	IL1B	−1.75	0.018			
PEA15	0.57	0.043	RPL13A	0.78	0.025			
PYCARD	0.86	0.049	IRF1	0.78	0.034			
CCL2	−2.92	0.053	CCL2	−2.92	0.053			
RPL13A	0.65	0.056	CCL5	−1.06	0.053			

*Tc* vs control at 18h			*Tc* + ATP vs control at 18 h			*Tc* + ATP vs *Tc* at 18 h		
Gene name	ddCt log ratio	p value	Gene name	ddCt log ratio	p value	Gene name	ddCt log ratio	p value
CXCL1	−3.67	0.000	TNF	−3.97	0.000	NAIP	−4.79	0.001
TNF	−3.60	0.000	CXCL1	−3.35	0.000	MAPK12	1.40	0.011
HSP90B1	2.13	0.000	HSP90B1	2.41	0.000	CHUK	1.14	0.049
CXCL2	−4.07	0.000	CXCL2	−4.34	0.000	TIRAP	2.57	0.076
GAPDH	1.61	0.000	PYCARD	2.10	0.000			
MAPK1	1.40	0.000	MAPK1	1.21	0.000			
CTSB	1.86	0.000	GAPDH	1.39	0.001			
CCL5	−2.26	0.000	CCL5	−2.17	0.001			
NAIP	5.05	0.001	TAB2	1.20	0.001			
PYCARD	1.64	0.001	CTSB	1.56	0.001			
SUGT1	1.21	0.003	MAP3K7	1.15	0.002			
PSTPIP1	1.16	0.004	MAPK12	1.75	0.003			
TRAF6	1.66	0.005	PSTPIP1	1.14	0.005			
MAPK3	1.26	0.012	CHUK	1.70	0.006			
TAB2	0.81	0.011	NFKBIA	−1.26	0.008			
CFLAR	−1.77	0.016	B2M	1.60	0.013			
IL1B	−1.79	0.016	P2RX7	−2.18	0.013			
MAP3K7	0.86	0.015	CASP4	0.79	0.018			
TNFSF14	1.60	0.016	NLRC5	3.65	0.016			
ACTB	1.60	0.025	PEA15	0.70	0.017			
CASP4	0.70	0.032	TNFSF14	1.57	0.018			
CCL2	−3.34	0.030	RPL13A	0.81	0.022			
HSP90AA1	1.12	0.027	ACTB	1.60	0.025			
IRAK1	−3.86	0.029	HSP90AA1	1.12	0.027			
P2RX7	−1.85	0.030	IRF1	−0.83	0.026			
RPL13A	0.75	0.032	PTGS2	−4.56	0.024			
PANX1	1.00	0.034	TIRAP	3.29	0.028			
PEA15	0.56	0.047	CARD6	2.02	0.031			
CARD6	1.79	0.052	CCL2	−3.32	0.031			
			IRAK1	−3.85	0.030			
			PANX1	0.98	0.035			
			SUGT1	0.81	0.035			
			TRAF6	1.12	0.040			
			MAPK3	0.91	0.055			

The 96-well RT Profiler Human Inflammasome PCR Arrays (SA Biosciences/Qiagen) were probed in triplicate with cDNA from THP-1 macrophages infected with *T. cruzi* (*Tc*) for 3 h or 18 h (with or without ATP) as described in Materials and Methods. The Ct values from qPCR data were analyzed by using open source HTqPCR v.1.7 software package (v.2.13). All array data were normalized by Quantile method and filtered to exclude genes that exhibited Ct values>35. The relative expression level of each target gene in treated cells was calculated using the formula, fold change  = 2-ΔΔCt, where ΔCt represents the Ct (sample) - Ct (control). LimmaCt in HT-qPCR package was employed for contrast analysis of all the groups included in experiment and identification of genes that were overall differentially expressed (p<0.05).

### Functional characterization of differentially expressed genes in *Tc*-infected cells

The top biological and molecular functional analysis of the differentially expressed gene datasets in *Tc*-infected mφs by Ingenuity iReport is presented in Table 2. In infected THP-1 mφs at 3 h pi (± ATP), 13–16 of the differentially expressed genes were indicative of an increase in inflammatory responses and control of macrophages' cell death (p<0.001, z score: −1.195 to −2.333, p<0.001). Exogenous addition of ATP had no significant effect on the top biological functions altered in response to *T. cruzi* infection. Toxicity analysis indicated that changes in mitoChondrial membrane potential that would likely result in ROS generation would be a key event in inflammatory and cell remodeling/cell death responses (p<0.01). By 18 h pi (± ATP), 14–21 of the differentially expressed genes were involved in inflammation, activation of leukoCytes, and/or migration of phagoCytes (z score: 1.78 to 2.6) and 24–26 of the differentially expressed genes implicated in cell death were down-regulated (z score: −1.23 to −1.25). The top canonical pathway, toxicity, and upstream regulators analysis suggested the macrophage's attempt to balance inflammation with cellular protection via PPAR-α signaling (p<0.001).

**Table 2:** Ingenuity iReport analysis of inflammasome-related datasets in THP-1 macrophages infected by *T. cruzi* for 3 h or 18 h (± ATP).

Pathways	Gene name	FoCus mols	P value
***T. cruzi*** ** infection of THP-1 mφs vs control (3h)**			
***Top biological and molecular functions***			
1) Inflammatory responses/infection	↓B2M, ↓BCL2, ↑CCL2, ↑CCL5, ↓CIITA, ↑CXCL1, ↑CXCL2, ↑NFKB1, ↑NFKB1A, ↓PYCARD, ↑RIPK2,↑TNF, ↓TXNIP	13	5.91E-12–2.49E-04
1a. Recruitment of neutrophils/phagoCytes	↑CCL2, ↑CCL5, ↑CXCL1, ↑CXCL2, ↓PYCARD, ↑RIPK2, ↑TNF	7	2.77E-10
1b. Activation of leukoCyte/lymphoCytes	↓BCL2, ↑CCL2, ↑CCL5, ↑CXCL1, ↑CXCL2, ↑NFKB1, ↑NFKB1A, ↓PYCARD, ↑TNF	9	1.89E-07
2) Cell death of immune cells (decreased)	↓B2M, ↓BCL2, ↑CCL2, ↑CCL5, ↓CIITA, ↑CXCL1, ↑CXCL2, ↓MAPK1, ↑NFKB1, ↑NFKB1A, ↓PEA15, ↓PYCARD, ↑RIPK2, ↓TAB2, ↑TNF, ↓TXNIP	16	4.50E-10
2a. Macrophage cell death (decreased)	↓BCL2, ↑CCL2, ↑CCL5, ↑CXCL1, ↑CXCL2, ↑NFKB1, ↑NFKB1A, ↓PYCARD, ↑TNF	9	3.78E-13
***Toxicity analysis***			
Decreases MPT, mitoChondrial swelling	↓B2M, ↓BCL2, ↑NFKB1, ↓PYCARD, ↑TNF	5	7.27E-08
***T. cruzi*** ** infection of THP-1 mφs vs control (3 h + ATP)**			
***Top biological and molecular functions***			
1) Inflammatory responses/infection	↓BCL2, ↑CCL2, ↑CCL5, ↑CXCL1, ↑CXCL2, ↓IL1B, ↓IRF1,↑NFKB1, ↑NFKB1A, ↓NLRP3, ↑PTGS2, ↓PYCARD, ↑RIPK2,↑TNF, ↓TXNIP	16	7.54E-14–1.79E-05
1a. Recruitment/migration of phagoCytes/neutrophils	↑CCL2, ↑CCL5, ↑CXCL1, ↑CXCL2, ↓IL1B, ↑MAPK3, ↑NLRP3, ↓PYCARD, ↑RIPK2, ↑TNF	9	7.69E-09
1b. Activation of lymphoCyte/leukoCytes	↓BCL2, ↑CCL2, ↑CCL5, ↑CXCL1, ↓IL1B, ↓IRF1, ↑NFKB1, ↑PTGS2, ↓PYCARD, ↑RIPK2 ↑TNF, ↓TXNIP	12	3.44E-12
2) Cell death of immune cells/necrosis (decreased)	↓BCL2, ↑CCL2, ↑CCL5, ↑CXCL1, ↑CXCL2, ↓IL1B, ↓IRF1, ↑MAPK3, ↑NFKB1, ↑NFKB1A, ↑NLRP3, ↑PTGS2, ↓PYCARD, ↑RIPK2, ↑TNF, ↓TXNIP	16	9.06E-17–1.99E-05
2a. Cell death of phagoCytes, myeloid cells (decreased)	↓BCL2, ↑CCL2, ↑CCL5, ↑CXCL1, ↑CXCL2, ↓IL1B, ↓IRF1, ↑NFKB1, ↑NFKB1A, ↓PYCARD, ↑TNF	11	9.06E-17
***Toxicity analysis***			
Decreased MPT, mitoChondrial swelling	↓B2M, ↓BCL2, ↑NFKB1, ↓PYCARD, ↑TNF	5	7.27E-08
***T. cruzi*** ** infection of THP-1 mφs vs control (18 h)**			
***Top biological and molecular functions***			
1) Cell death of immune cells (decreased)	↓ACTB, ↓CASP4, ↑CCL2, ↑CCL5, ↑CFLAR, ↓CTSB, ↑CXCL1, ↑CXCL2, ↓GAPDH, ↓HSP90AA1, ↓HSP90B1, ↑ IL1B, ↑IRAK1, ↓MAP3K7, ↓MAPK1, ↓MAPK3, ↓NAIP, ↑P2RX7, ↓PEA15, ↓PYCARD, ↓TAB2, ↑TNF, ↓TNFS14, ↓TRAF6	24	1.48E-12–2.60E-04
1a.Cell death of myeloid/phagoCytes	↓CASP4, ↑CCL2, ↑CCL5, ↑CFLAR, ↑CXCL1, ↑CXCL2, ↑ IL1B, ↑P2RX7, ↓PYCARD, ↑TNF	10	1.72E-12
2) Inflammation/infectious disease	↓CASP4, ↑CCL2, ↑CCL5, ↑CXCL1, ↑CXCL2, ↓HSP90AA1, ↑ IL1B, ↓MAPK1, ↓PANX1, ↑P2RX7, ↓PYCARD, ↑TNF, ↓TNFS14, ↓TRAF6	14	1.08E-06
2a. Activation of leukoCyte/lymphoCytes	↑CCL2, ↑CCL5, ↑CXCL1, ↓HSP90B1, ↓IL1B, ↓MAP3K7, ↓PYCARD, ↑TNF, ↓TNFS14	9	2.95E-07
2b. Migration of phagoCytes/neutrophils	↑CCL2, ↑CCL5, ↓CTSB,↑CXCL1, ↑CXCL2, ↑IL1B, ↑P2RX7, ↑TNF	8	2. 07E-07
***Toxicity analysis***			
Gene regulation by PPARα	↓HSP90AA1, ↑IL1B, ↓MAP3K7, ↓MAPK1, ↓MAPK3, ↑TNF, ↓TRAF	7	4.16E-10
***T. cruzi*** ** infection of THP-1 mφs vs control (18 h + ATP)**			
***Top biological and molecular functions***			
1) Cell death (decreased)	↓ACTB, ↓B2M, ↓CASP4, ↑CCL2, ↑CCL5, ↓CHUK, ↓CTSB,↑CXCL1, ↑CXCL2,↓GAPDH, ↓HSP90AA1, ↓HS90B1, ↑IRAK1, ↑IRF1, ↓MAP3K7, ↓MAPK1 ↓MAPK12, ↑NFKB1A, ↑P2RX7, ↓PEA15, ↑PTGS2, ↓PYCARD, ↓TAB2, ↑TNF, ↓TNFS14, ↓TRAF6	26	6.53E-12–4.09E-04
1a.Cell death of myeloid/phagoCytes	↓CASP4, ↑CCL2, ↑CCL5, ↑CFLAR, ↑CXCL1, ↑CXCL2, ↑ IL1B, ↑P2RX7, ↓PYCARD, ↑TNF	10	1.48E-12
2) Inflammation/Infectious disease	↓ACTB, ↓B2M, ↓CASP4, ↑CCL2, ↑CCL5,↓CHUK, ↓CTSB, ↑CXCL1, ↑CXCL2, ↓HSP90B1, ↑IRAK1 ↓MAP3K7, ↑NFKB1A, ↑PTGS2, ↑P2RX7, ↓PYCARD, ↓TIRAP, ↑TNF, ↓TNFS14, ↓TRAF6	21	3.33E-10–4.43E-04
2a. Activation of leukoCyte/lymphoCytes	↓B2M, ↑CCL2, ↑CCL5, ↑CXCL1, ↑CXCL2, ↓MAP3K7, ↑TNF	7	9.31E-06
2b. Migration of phagoCytes/neutrophils	↑CCL2, ↑CCL5, ↓CHUK, ↓CTSB, ↑CXCL1, ↑TNF ↑CXCL2, ↑NFKB1A, ↑P2RX7, ↑PTGS2, ↓TIRAP	11	2.43E-04
***Toxicity analysis***			
Gene regulation by PPARα	↓CHUK, ↓HSP90AA1, ↓MAP3K7, ↓MAPK1, ↓MAP3K7, ↑NFKB1A, ↑PTGS2, ↑TNF, TRAF6	9	2.11E-11

All differentially expressed proteins identified in THP-1 macrophages infected with *T. cruzi* for 3 or 18 h (±ATP) (listed in Table 1) were uploaded into Ingenuity Pathway Analysis (IPA) to interpret datasets in the context of biological proCesses and function, and pathway and molecular networks. Presented are the top networks with a p value <0.01 to which maximal number of the differentially expressed proteins identified in chagasic plasma (bolded letters) were assoCiated with. FoCus molecules are the number of differentially expressed plasma proteins assoCiated with an individual network.

### Caspase-1-mediated IL-1β activation is ROS-dependent, and plays a role in control of *T. cruzi* replication in wild-type mφs

**Figure 3:**
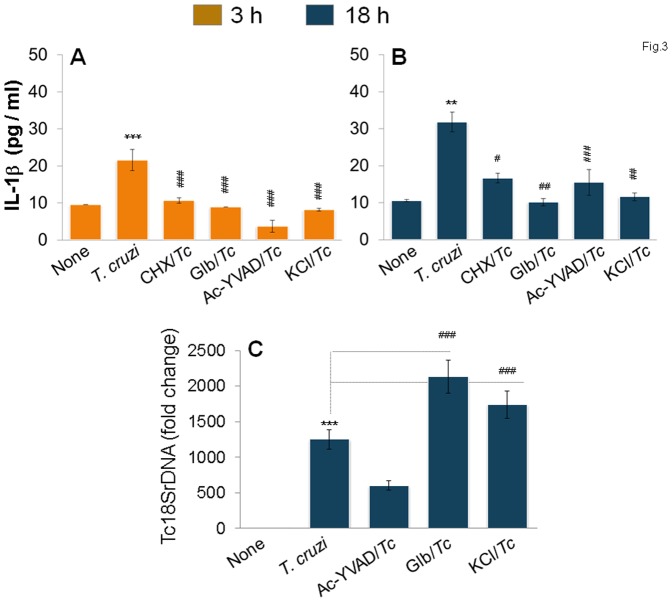
NLRP3/caspase-1 inflammasome is the major source of IL-1β for parasite control in mφs. THP-1 mφs were incubated with *T. cruzi* in the presence or absence of cycloheximide (CHX,), glibenclamide (Glb), Ac-YVAD-CHO, and KCl for 3 h *(A)* and 18 h *(B&C)*. Macrophages incubated with media alone were used as controls. *(A&B)* IL-1β release in supernatants was determined by an ELISA. *(C)* Quantitative PCR analysis of parasite burden in infected macrophages using *Tc18SrDNA*-specific oligonucleotides.

To determine if inflammasomes play a role in IL-1β activation in mφs infected by *T. cruzi*, we pre-treated the THP-1 mφs with selective inhibitors of inflammasome activation for 2 h and continued the inhibition pressure during the infection period. The IL-1β release induced at 3 h and 18 h pi was decreased by 100% and 75–95%, respectively, when mφs were treated with cycloheximide (inhibits protein synthesis and thus the inducible arm of the inflammasome pathway), glibenclamide (bloCks the caspase-1 and pro-IL-1β maturation), Ac-YVAD-CHO (inhibits caspase-1 activity), or KCl (inhibits K^+^ efflux required for caspase 1 activation) (Fig.3A&B). Importantly, glibenclamide and KCl also bloCked the mφs' ability to control parasite replication, resulting in a 50–70% increase in intracellular Tc18SrDNA, as determined by sensitive qPCR (Fig.3C).

**Figure 4:**
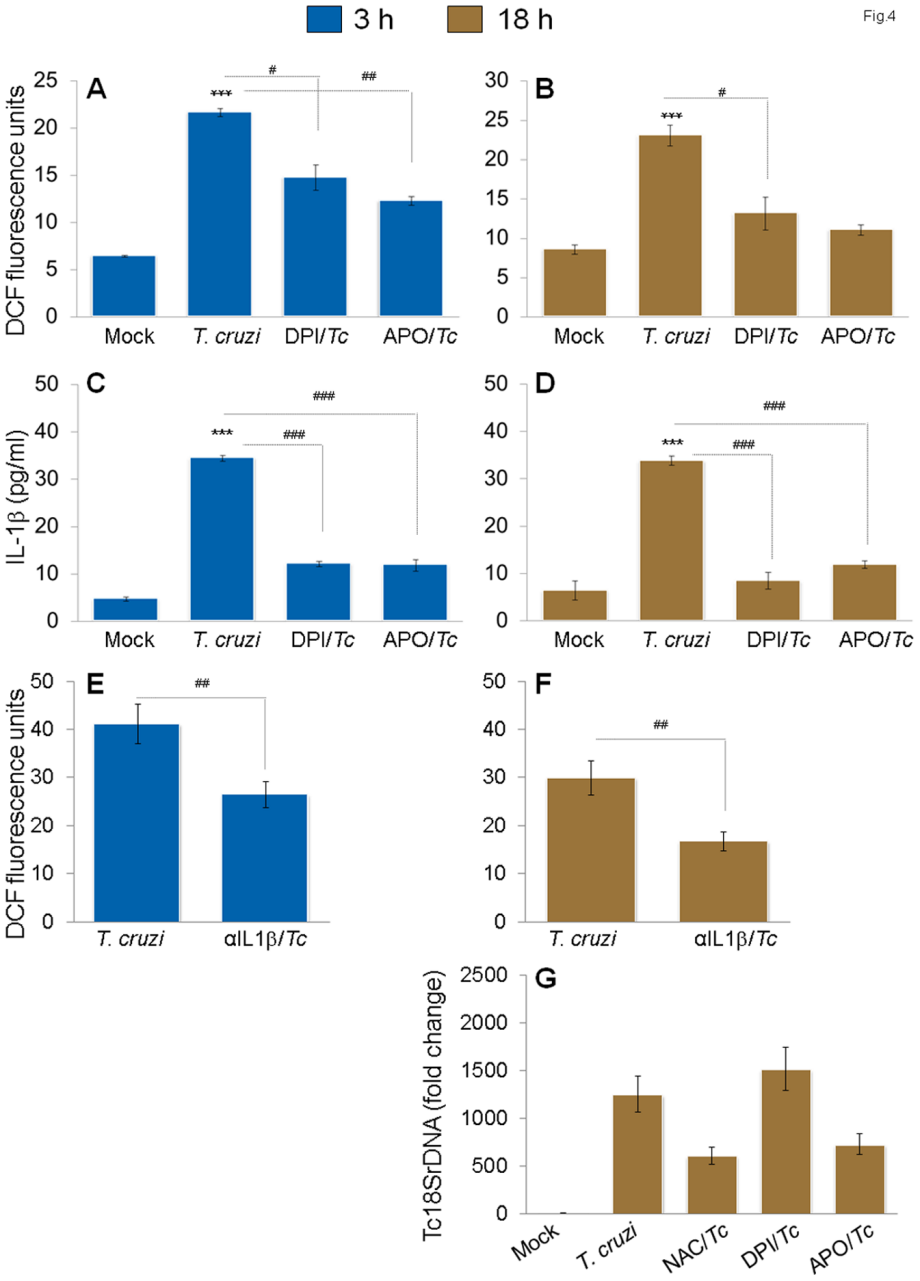
Feedback cycle of NOX2/ROS and IL-1β activation in mφs infected by *T. cruzi*. THP-1 mφs were infected with *T. cruzi* as in Fig. 1, and incubated for 3 h *(A,C,E)* or 18 h *(B,D,F,G)* in presence of NOX2 inhibitors (diphenylene iodinium (DPI) or apoCynin), ROS scavenger (N-acetylcysteine (NAC)) or IL-1β antibody. *(A*–*D)* NOX2 inhibitors decreased ROS and IL-1β levels in infected mφs. Shown are *(A&B)* H_2_DCFDA oxidation by intracellular ROS, resulting in formation of fluorescent DCF by fluorimetry and *(C&D)* IL-1β release in supernatants determined by an ELISA. *(E&F)* Treatment with anti-IL-1β antibody decreased the ROS levels in infected mφs. *(G)* Effect of ROS inhibitors on intracellular parasite burden, as determined by qPCR, in infected mφs.

Ingenuity iReport analysis of differentially expressed genes in this study (Table 2) and our previously published reports [22] have led us to suggest that infection by *T. cruzi* would elicit ROS by changes in mitoChondrial MPT or NOX2 activation in mφs, and that ROS may serve as a 2^nd^ signal for inflammasome activation [11], [23]. We, therefore, determined whether ROS is induced and plays a direct role in IL-1β production in infected mφs. THP-1 mφs exhibited 3.4-fold and 2.7-fold increases in DCF fluorescence (detects intracellular ROS) at 3 h and 18 h pi, respectively (Fig.4A). When NOX2 inhibitors (DPI or apoCynin) were added during incubation with *T. cruzi*, we noted a 21–44% and 68–80% decline in ROS (Fig.4A&B) and 37% and 45% decline in IL-β release (Fig.4C&D) at 3 h and 18 h pi, respectively. These data suggested that NOX2, at least partially, regulates ROS-dependent IL-1β activation in infected mφs. The observation of a moderate, but a significant (35–45%, p<0.05) decline in DCF fluorescence in infected mφs treated with anti-IL-1β antibody (Fig.4E&F) implied that IL-1β also contributes to activation of ROS production. However, we found no increase in intracellular parasite burden in THP-1 mφs treated with NOX2 inhibitors (Fig.4G). Together, the data presented in Figs.3&4 suggest that a feed-back cycle of caspase-1/IL-1β and ROS activation oCcurs in response to *T. cruzi* infection in THP-1 mφs and is required for control of intracellular parasite replication. While direct inhibition of IL-1β (Fig.1F) or caspase-1 (Fig.3C) affected the WT mφs ability to control *T. cruzi*, inhibition of ROS-dependent IL-1β was compensated for, likely by activation of other immune defenses capable of controlling the intracellular parasite replication and survival (Fig.4G).

### IL-1β signaling of NFκB in *Tc*-infected mφs

**Figure 5:**
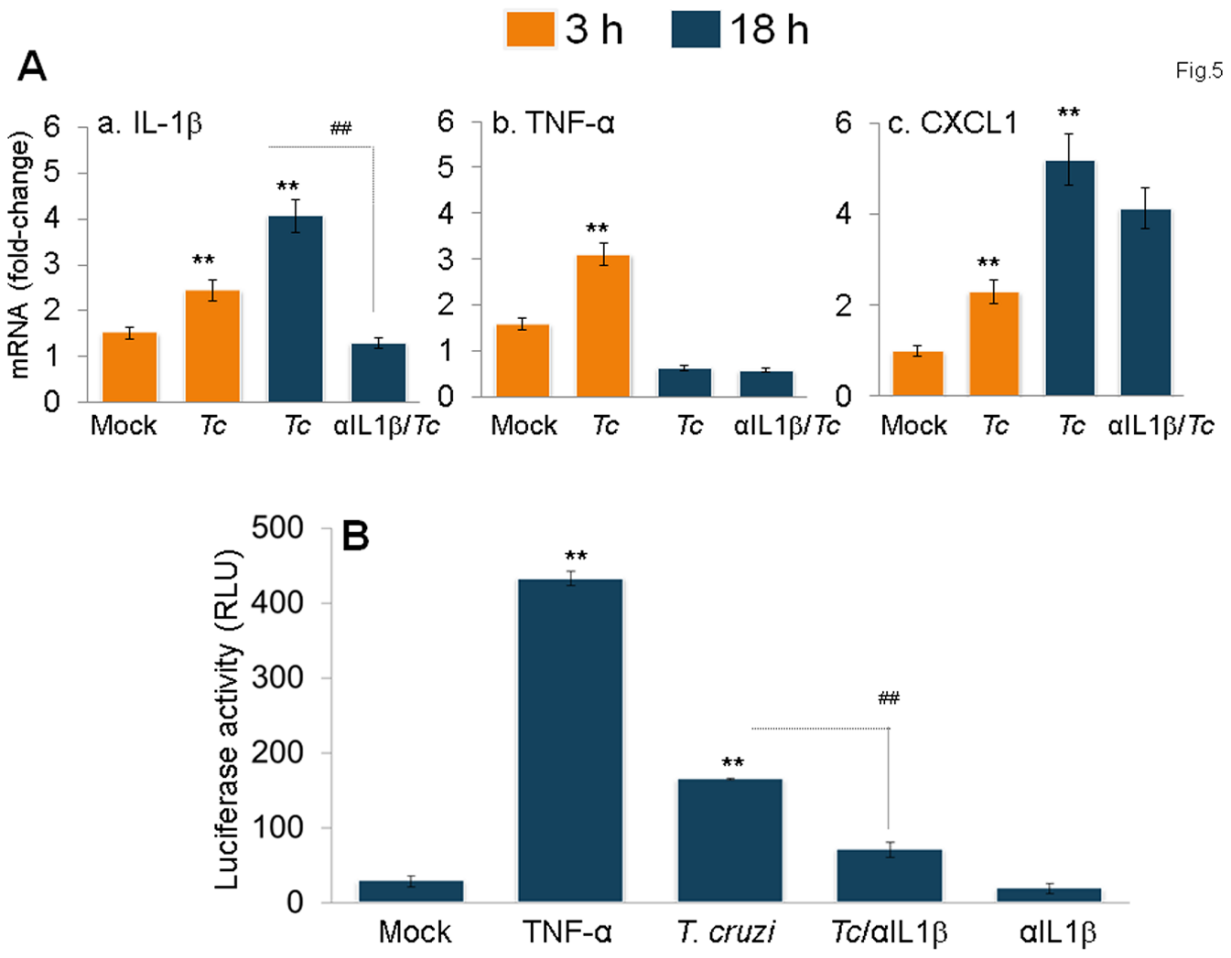
IL-1β signals NF-κB activation and inflammatory cytokine gene expression in infected mφs. *(A)* The mRNA levels for IL-1β (panel a), TNFα (panel b) and CXCL1 (panel c) cytokines were measured in *T. cruzi-*infected THP-1 mφs at 3h and 18h pi by quantitative RT-PCR. *(B)* RAW 264.7 macrophages were transiently transfected with pGL4.NF-κB-Luc reporter plasmid and pREP7-Rluc plasmid (transfection efficiency control) as described in Materials and Methods. Transfected cells were infected with *T. cruzi* and incubated in the presence or absence of anti-IL-1β antibody. Mφs incubated with 10 ng/ml recombinant TNF-α for 6 h were used as positive controls. The relative NF-κB transcriptional activity was measured by firefly luciferase activity and normalized to *Renilla* luciferase activity. The transcriptional activity of NF-κB in normal cells was considered as baseline and valued at 1.

Because gene expression analysis has identified several of the signaling (e.g. TRAF6, MYD88, NFKBA) and cytokine (e.g. CCL2, CCl5, CXCL1, TNF) molecules involved in inflammatory responses that were activated in *Tc*-infected mφs (Tables 1 & 2), we determined if IL-1β signals the activation of the nuclear factor κB (NF-κB) pathway of cytokine gene expression in mφs. The mRNA levels for IL-1β, TNF-α and CXCL1 in THP-1 mφs were increased by 1.6 fold, 2-fold, and 2.3-fold, respectively, at 3 h pi. At 18 h pi, IL-1β and CXCL1 were increased by 1.6-fold and 5-fold, respectively, while no increase was observed in TNF-α mRNA level (Fig.5A.a–c). Treatment of infected mφs with anti-IL-1β antibody abolished the increase in IL-1β mRNA in infected mφs; possibly indicating that the IL-1β engagement of surface receptors induced the IL-1β mRNA expression.

We performed a luciferase reporter assay to verify the role of IL-1β in signaling cytokine gene expression via NFκB in *Tc*-infected mφs. RAW mφs transiently transfected with luciferase reporter plasmid pNF-κB-luc with 3×NF-κB binding site and pREP7-Rluc (expresses renilla luciferase) were infected with *T. cruzi* for 18 h, and NF-κB-dependent luciferase activity (normalized to renilla luciferase) was monitored. We observed a >5-fold and 15-fold increase in luciferase activity, respectively, when mφs were infected with *T. cruzi* or treated with recombinant TNF-α (Fig.5B). Treatment with anti-IL-1β antibody resulted in 70% decline in *Tc*-induced luciferase activity (Fig.5B, p<0.01). Together, the data presented in Fig.5 suggested that IL-1β signals the NF-κB activation of cytokine gene expression in mφs infected by *T. cruzi*.

### ASC^-/-^ mφs were compromised in ROS-dependent IL-1β activation and NF-κB-dependent cytokine gene expression, and exhibited pronounced *T. cruzi* replication and survival

**Figure 6:**
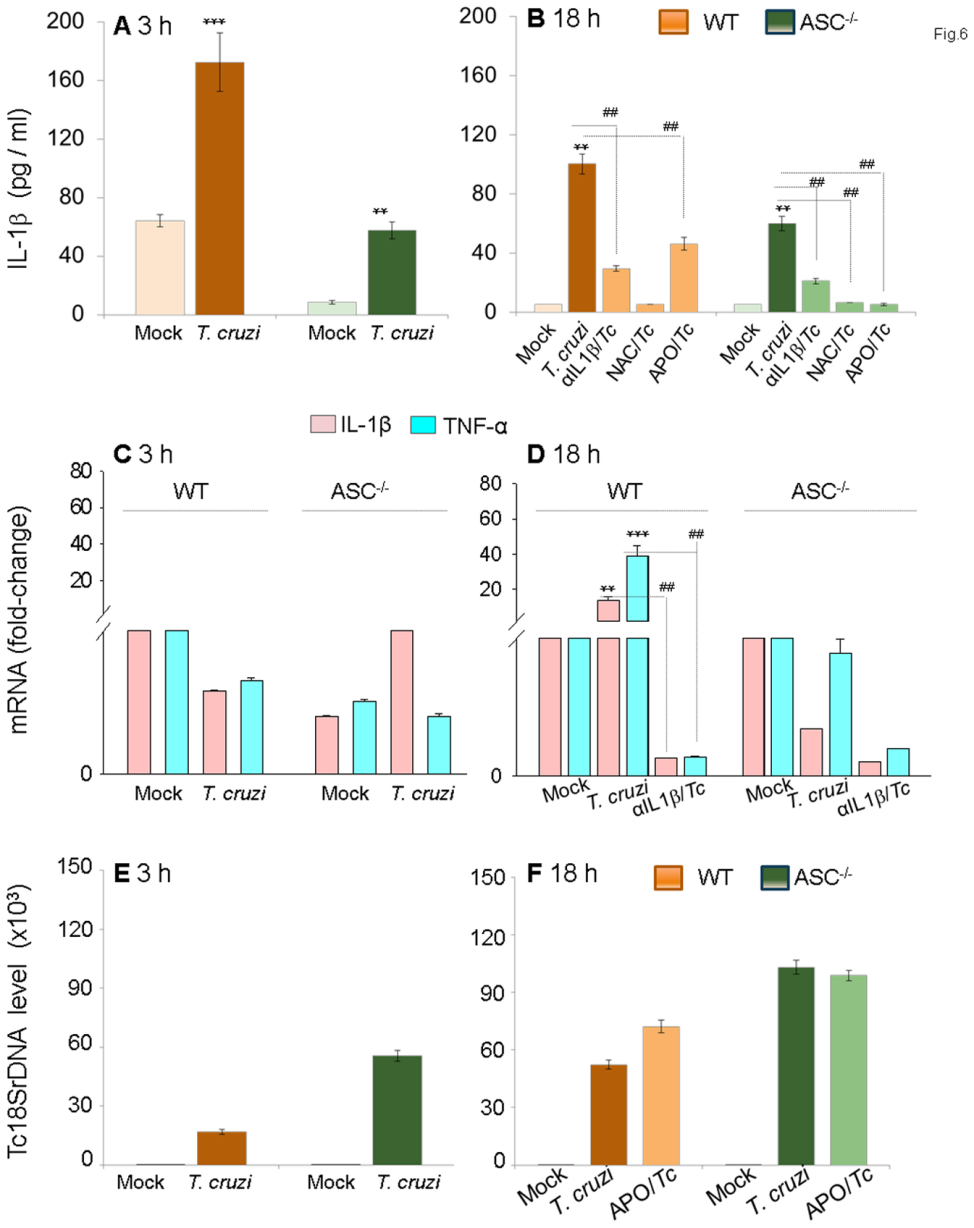
ASC^-/-^ mφs are compromised in the IL-1β–ROS–NF-κB pathway for control of *T. cruzi*. Bone-marrow-derived macrophages were isolated from matched WT and ASC^-/-^ mice. Primary mφs were infected with *T. cruzi* and incubated for 3 h or 18 h in the presence or absence of anti-IL-1β Ab or ROS scavengers (as in Figs.1&4). Shown are IL-1β release measured by an ELISA *(A&B)*, mRNA levels for IL-1β and TNF-α by quantitative RT-PCR *(C&D)* and Tc18SrDNA signal by qPCR *(E&F)*.

To determine if ASC is involved in caspase-1-dependent inflammasome formation and activation of inflammatory proCesses for parasite control, we utilized primary BM-derived mφs from ASC^-/-^ mice and matched controls. The ASC^-/-^ mφs infected by *T. cruzi* exhibited a significantly compromised IL-1β release (67% and 40% decline at 3 h and 18 h, respectively) when compared to that noted in matched WT controls (Fig.6A&B, p<0.01). Treatment with anti-IL-1β antibody and ROS scavengers (NAC or apoCynin) normalized the IL-1β production in *Tc*-infected ASC^-/-^ and WT mφs to control levels. Further, the expression of NF-κB-inducible pro-IL-1β and TNF-α mRNAs, that were significantly increased in *Tc-*infected WT mφs, were completely abolished in *Tc*-infected ASC^-/-^ mφs (Fig.6C&D). Subsequently, ASC^-/-^ mφs exhibited a 3.2-fold and 2-fold increase in intracellular parasite burden at 3 h and 18 h pi, respectively, when compared to that noted in matched WT infected mφs (Fig.6E&F). As previously observed in THP-1 mφs (Fig.4G), ASC^-/-^ mφs exhibited no further increase in parasite burden upon treatment with ROS scavengers (Fig.6). These data confirmed that the caspase-1/ASC inflammasomes play an important role in parasite control through ROS-dependent IL-1β activation and expression of other inflammatory cytokines in mφs.

### NLRP3^-/-^ mφs exhibited increased ROS-dependent control of *T. cruzi*

**Figure 7:**
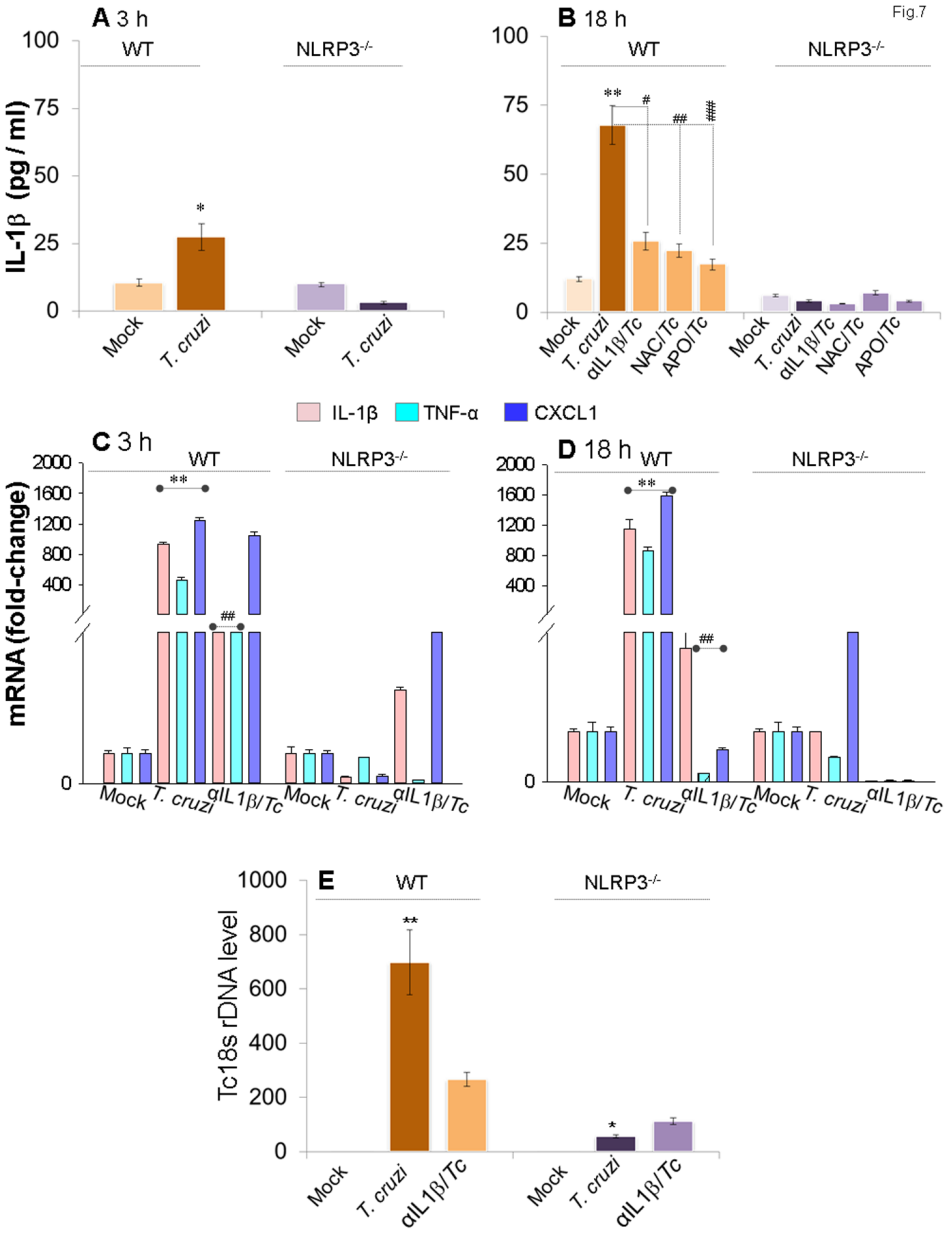
NLRP3^-/-^ mφs are compromised in IL-1β activation and inflammatory cytokine gene expression, but equipped to control *T. cruzi*. Bone marrow-derived macrophages were isolated from matched WT and NLRP3^-/-^ mice. Primary mφs were infected with *T. cruzi* and incubated for 3 h or 18 h in the presence or absence of anti-IL-1β antibody or ROS scavengers (as in Figs.1&4). Shown are IL-1β release by ELISA *(A&B)*, mRNA level for IL-1β, TNF-α and CXCL1 by quantitative RT-PCR *(C&D)* and *Tc*18SrDNA signal by qPCR *(E)*.

To specifically determine if NLRP3 inflammasome activation by ASC/caspase-1 is the primary source of protective IL-1β in the context of *Tc* infection, we conducted further studies in primary bone marrow-derived mφs from NLRP3^-/-^ mice and matched controls. As expected from studies in ASC^-/-^ cells, NLRP3^-/-^ mφs lacked the ability to elicit IL-1β activation in response to *T. cruzi* infection (Fig.7A&B, p<0.01). Treatment with anti-IL-1β antibody and ROS scavengers (NAC or apoCynin) normalized the IL-1β production in *Tc*-infected WT mφs to control levels (Fig.7B). Further, NF-κB-inducible pro-IL-1β, TNF-α and CXCL1 mRNA levels that were increased in *Tc-*infected WT mφs, were almost absent in *Tc*-infected NLRP3^-/-^ mφs (Fig.7C&D). However, to our surprise, NLRP3^-/-^ mφs exhibited a 12-fold decline in intracellular parasite burden at 18 h, when compared to that noted in matched WT infected mφs (Fig.7E).

**Figure 8:**
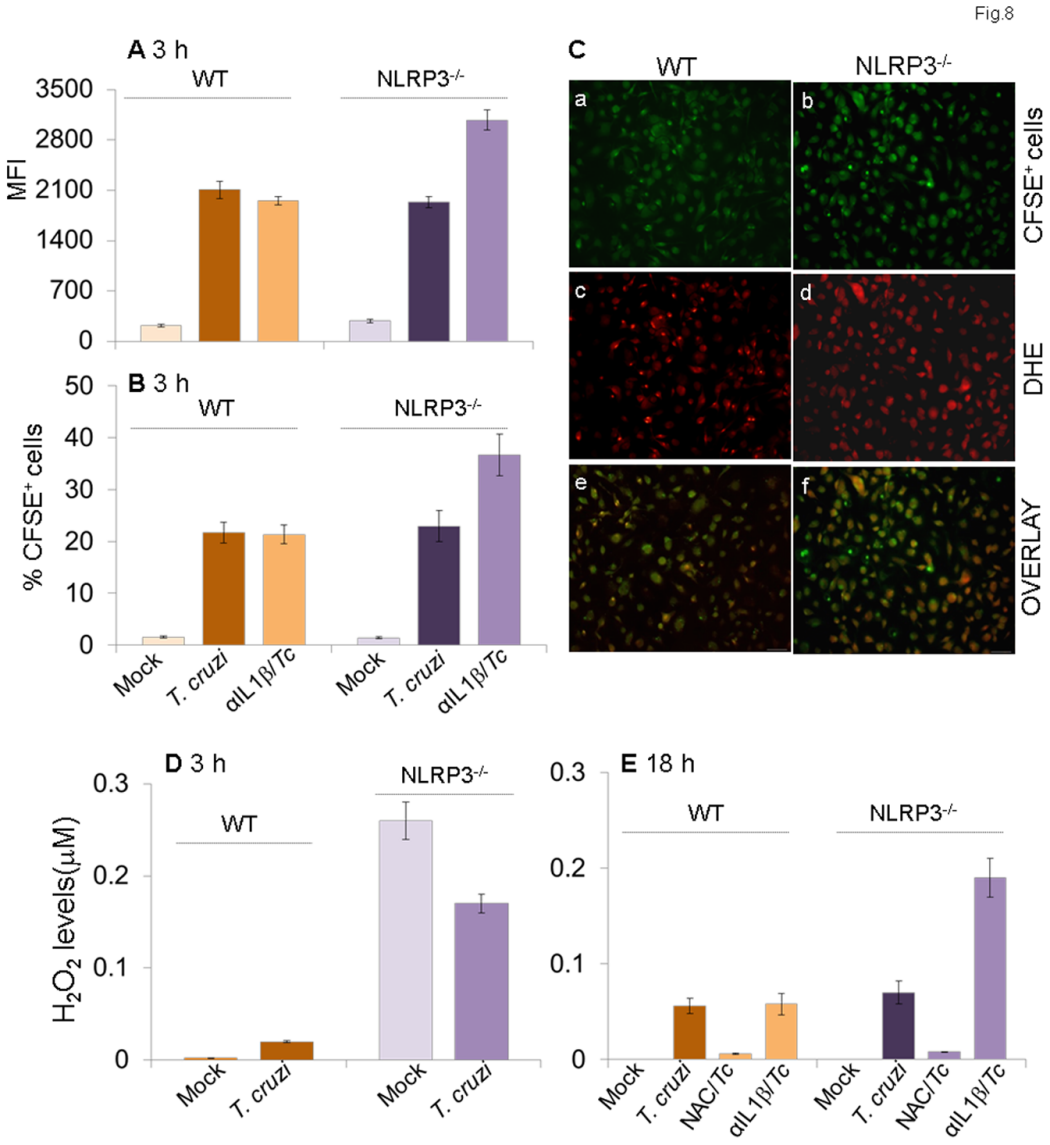
NLRP3 deficiency is compensated for by increased ROS levels in mφs (± *T. cruzi*). Bone marrow-derived primary macrophages isolated from matched WT and NLRP3^-/-^ mice were infected with CFSE-labeled *T. cruzi* and incubated for 3 h or 18 h in the presence or absence of anti-IL-1β antibody or ROS inhibitors. Shown are the mean fluorescence intensity of CFSE *(A)* as an indicator of # parasites/cell and mean percentage of CFSE^+^ mφs *(B)* as an indicator of parasite uptake efficiency. *(C)* Fluorescence microscopy of NLRP3^-/-^ (panels a, c, e) and WT (panels b, d, e) mφs infected with CFSE-labeled *T. cruzi* for 18 h. Shown are representative images of CFSE (green, panels a & b), intracellular ROS-specific DHE fluorescence (panels c & d) and overlay images of a & c and b & d in panels e & f. *(D&E)* Bar graphs show a quantitative measure of ROS release, measured by an Amplex red assay, in NLRP3^-/-^ and WT mφs.

To determine if NLRP3^-/-^ mφs were simply refractory to *T. cruzi*, we incubated the NLRP3^-/-^ and matched WT mφs with CFSE-labeled trypomastigotes for 3 h and analyzed for CFSE fluorescence by flow cytometry. We observed no statistically significant difference in intracellular mean fluorescence intensity (MFI) indicative of the number of parasites/cell (Fig.8A) and the percentage of CFSE^+^ cells indicative of the number of *Tc*-infected NLRP3^-/-^ and WT mφs (Fig.8B). Representative micrographs showing efficient uptake of CFSE-labeled parasites in NLRP3^-/-^ and WT primary mφs are presented in Fig.8C (panels a&b). These data suggested that NLRP3^-/-^ mφs were not refractory to *T. cruzi* and efficiently phagoCytized parasites in a manner similar to that of the WT mφs. Instead, NLRP3^-/-^ mφs, in comparison to WT mφs, exhibited 8.5-fold higher basal ROS levels (Fig.8D). The ROS production in NLRP3^-/-^ mφs was enhanced upon *T. cruzi* infection and not inhibited by the addition of anti-IL-1β antibody (Fig.8D&E). The increase in ROS production in NLRP3^-/-^ mφs in response to *T. cruzi* infection was also evidenced by a significant increase in DHE fluorescence (red, detects intracellular ROS, Fig.8C.c–f). Together, the data presented in Figs.7&8 suggested that NLRP3/ASC/caspase-1 inflammasome mediates IL-1β activation and expression of other inflammatory cytokines in mφs infected by *T. cruzi*; however, NLRP3 deficiency is compensated for by increased ROS levels capable of preventing parasite replication and intracellular survival in NLRP3^-/-^ mφs.

## Discussion

The innate immune response to *T. cruzi* infection is mediated by diverse PRRs, including receptors of the TLR family [24], [25] and ASC-containing inflammasomes (e.g. NOD1 [26], and NLRP3 [27], [28]). Genetically modified mice deficient in MYD88 (interacts with TLR-2, -4, -6), TLR-4, NOD1, and ASC exhibited an increased susceptibility to *T. cruzi*, thus pointing to these PRRs as critical determinants of host resistance to *T. cruzi* infection [24], [26], [28], [29]. In this manuscript, we have utilized cultured and primary mφs and employed inhibitory approaches to investigate the mechanisms of caspase-1/ASC inflammasome activation and their role in the context of *T. cruzi* infection. We found that mφs respond to *T. cruzi* infection with suboptimal activation of inflammasome-related gene expression and IL-1β production. Functional analysis of the differentially expressed gene datasets in *Tc*-infected mφs was indicative of an increase in inflammatory responses and control of macrophages' cell death. The IL-1β production in *Tc*-infected mφs was ROS-dependent and could be enhanced by the exogenous addition of ATP. Studies in WT mφs treated with specific inhibitors and ASC^-/-^ mφs suggested that caspase-1/ASC inflammasome played a role in activation of the IL-1β–ROS–NF-κB pathway, that when inhibited, resulted in a compromised inflammatory cytokine response and increase in *T. cruzi* replication and survival in macrophages. However, NLRP3^-/-^ mφs, despite an inability to elicit IL-1β activation and inflammatory cytokine gene expression, were capable of parasite control. Thus, our data allow us to conclude that caspase-1/ASC inflammasomes play a significant role in the activation of IL-1β/ROS and NF-κB signaling of cytokine gene expression for *T. cruzi* control in human and mouse mφs. However, NLRP3 balances the mφs' activation of ROS and NFκB/cytokine response, and its deficiency shifted the mφs' responses towards increased ROS-dependent control of *T. cruzi*. To the best of our knowledge, this is the first study demonstrating a double-edged role of NLRP3 in determining macrophage activation of ROS and cytokine response, both of which are required for clearance of *T. cruzi* infection.

The NLRs are expressed in most cell types of the immune system, but are also reported to be expressed in other tissues. Based upon the expression profile of components of NLRP1, NLRP3, and NLRC1; blood, placenta and thymus are shown to constitutively express inflammasomes. Other tissues (e.g., heart, vascular tissue, bone marrow) require up regulation of one or two components in order to assemble functional inflammasomes [30]. Our data demonstrated that inflammasome-related gene expression is induced in mφs exposed to *T. cruzi* infection (Table 1) or LPS treatment (Table S2), as well as in non-phagoCytic, human vascular smooth muscle, fibroblast, and microvascular endothelial cells (Table S3). Functional analysis of the gene expression profile indicated that in mφs, an inflammatory response to control *T. cruzi* infection was assoCiated with significant efforts to prevent cell death (Table 2). Infected mφs also exhibited suppression of several of the genes involved in PPARα signaling that induces apoptosis following activation with TNF-α/IFN-γ [31], [32]. In non-phagoCytic cells, TLR/MYD88 signaling of NFκB-dependent cytokines' (IL-6, IFNα/β, IL-12) gene expression and caspase-1/NLRP3-mediated activation of IL-1β gene expression was noted. Canonical analysis, as in mφs, also indicated PPAR-α regulation of gene expression in HMVEC and HFF cells infected by *T. cruzi* (Table S4). Our observations allow us to surmise that phagoCytic and non-phagoCytic cells responded to *T. cruzi* infection by induction of diverse inflammasome-related gene expression. Eventually, all cell types appeared to be overwhelmed by the inflammatory signal and tended to switch to signaling events related to prevention of cell death.

Several studies indicate that ROS are an essential secondary messenger for signaling caspase-1/ASC inflammasome activation [11], [33]. The use of ROS scavengers controlled IL-1β activation by virtually all agonists of the NLRP3 inflammasome [33], [34]. Besides ROS, extracellular ATP, through activation of the P2X7 (purogenic ionotrophic ATP-gated cation channel), triggers K^+^ efflux, which, in turn, triggers pore formation by pannexin, thereby allowing the delivery of pathogen products into the cytosol, resulting in caspase-1/ASC inflammasome activation [35]. In agreement with the literature, we also observed ROS-dependent IL-1β activation mediated by caspase-1/ASC inflammasome in mφs exposed to *T. cruzi* (Figs.1–4). The exogenous addition of ATP resulted in a higher level of IL-1β activation in infected mφs at 3 h pi, as also noted by others [28]. The lack of an effect of exogenous ATP on the extent of IL-1β activation in infected mφs at 18 h pi may mean that other signals were generated. We speculate that either *Tc* kDNA released by dying *T. cruzi* within mφs or mtDNA released due to bystander damage in infected mφs served as a secondary signal in activating caspase-1/ASC inflammasomes at 18 h pi. This notion is supported by the observation of similar levels of IL-1β activation in mφs incubated with live as well as dead *T. cruzi* and requires further investigation.

Importantly, we made a novel observation and noted a feed-back cycle of IL-1β signaling of ROS activation in infected mφs (Figs.3&4). The molecular mechanism for the ROS production by IL-1β remains to be elucidated. In previous studies, IL-1β was found to stimulate phospholipase A_2_, promoting release of arachidonic acid. Since arachidonic acid can activate NAPDH oxidase to produce superoxide, it is possible that this fatty acid may serve as an intermediate in the IL-1β-induced activation of enzymes, leading to the production of ROS [36], [37]. However, several non-NADPH oxidase-dependent sources, including mitoChondrial electron transport and arachidonate metabolism, may also be involved in the cytokine-induced ROS generation, to be investigated in future studies.

It is intriguing that both ASC^-/-^ and NLRP3^-/-^ mφs were equally invaded by *T. cruzi*, lacked IL-1β production, and elicited ineffective, NF-κB-mediated cytokine gene expression (Figs.6–8); yet only ASC^-/-^ mφs were restrictive in their capacity to control *T. cruzi* infection (Fig. 6). ASC^-/-^ and caspase-1^-/-^ mice have been doCumented to exhibit a higher incidence of mortality, cardiac parasitism and heart inflammation, meaning that ASC/caspase-1 inflammasomes are critical determinants of host resistance to infection with *T. cruzi* [28]. Like ASC^-/-^ mice and *in vitro* cultured ASC^-/-^ mφs, NOD1^-/-^ mice and BM-derived derived mφs from NOD1^-/-^ mice also showed an impaired induction of NF-κB-dependent products and failed to restrict *T. cruzi* infection [26]. Our finding that a deficiency of NLRP3 did not affect mφs ability to control parasites allows us to surmise that the caspase-1/ASC requirement for effective control of *T. cruzi* is delivered via formation and activation of inflammasomes with other NLRs, e.g., NLRP1, AIM2 or NLRC4. The ability of NLRP3^-/-^ mφs to efficiently manage parasite killing via enhanced ROS release (Figs.7&8) suggest that NLRP3 suppresses NOX2-dependent ROS production in mφs. Though it is shown that activation of NLRP3 containing inflammasome is not dependent on the function of NOX1-4 [38], our data provide the first evidence that NLRP3 dysregulates the NOX2 function at the superoxide production level and suppresses the mφs' ability to kill *T. cruzi*. Caspase-l, upon activation by NLRP3/ASC, loCalizes to phagosomes and disturbs NOX2 control of pH, thereby triggering acidification and microbicidal activity of phagosomes in mφs infected by *StaphyloCoCcus aureus* [39]. We surmise that caspase-1 loCalization to phagosome and suppression of NOX2/ROS enhances the bactericidal activity of mφs. However, NLRP3 interaction with NOX2 resulting in low level of ROS production likely maintains the alkalization of the phagosomal lumen that prevents the mφs' ability to directly kill *T. cruzi*, but plays a critical role in allowing mφs to function as specialized phagoCytes adapted to proCess antigens for cross presentation and elicitation of adaptive immunity.

Other investigators have shown a severe defect in nitric oxide (NO) production and impairment in mφ-mediated *T. cruzi* killing in NLRP3^-/-^ mice and isolated mφs [27]. In this study, we utilized the BM-derived monoCytes from NLRP3^-/-^ mice that were differentiated to mφs by M-CSF treatment, and infected with *T. cruzi* (SylvioX10) for 3 h and 18 h. Others utilized peritoneal mφs obtained 4 days after intra-peritoneal injection of 1% starch solution in NLRP3^-/-^ mice and infected these mφs with the Y strain of *T. cruzi* for 48 h for all the studies [27]. We propose that NLRP3 deficiency is compensated for by over activation of NOX2/ROS that effectively controlled the early invasion and replication of *T. cruzi* in mφs, as observed in this study. However, a lack of NF-κB-mediated cytokine response (Fig.7) and iNOS/NO activation [27] prevented the NLRP3^-/-^ mφs from sustained, long-term control of the parasites, resulting in increased susceptibility. It is also plausible that the differences in the source of mφs, parasite isolates, and the time course of infection may explain the observed differences in the ability of NLRP3^-/-^ mφs to control *T. cruzi* infection in our and published studies.

In summary, we have demonstrated that *T. cruzi* interfered with the potent activation of caspase-1/ASC inflammasome-related gene expression and cytokine response in mφs to ensure its survival. We found that caspase-1/ASC inflammasomes played a role in the activation of the IL-1β–ROS–NF-κB pathway, that when inhibited, resulted in an increase in *T. cruzi* replication and survival in mφs. However, NLRP3^-/-^ mφs were compensated for by increased NOX2/ROS activation capable of parasite killing. Our data suggest that the NLRP3/caspase/ASC inflammasome balances the mφs' activation of ROS and NFκB/cytokine response and provide the first evidence for NLRP3 regulation of NOX2 function as an effector mechanism contributing to parasite persistence.

## Supporting Information

Table S1 **Oligonucleotides used in this study.**
(DOCX)

Table S2 **Inflammasome-related differential gene expression in THP-1 macrophages in response to LPS treatment (± ATP) in comparison to normal controls.**
(DOC)

Table S3 **Inflammasome-related differential gene expression in non-phagoCytes at 24 h infection by** ***Trypanosoma cruzi*** **.**
(DOCX)

Table S4 **Ingenuity iReport analysis of inflammasome-related datasets in non-phagoCytes infected by** ***T. cruzi*** **.**
(DOCX)

## References

[1] World Health Organization (2010) Chagas disease: control and elimination. UNDP/World Bank/WHO. Available: http://apps.who.int/gb/ebwha/pdf_files/WHA63/A63_17-en.pdf. Accessed 2014 Sept 3

[2] Machado FS, Dutra WO, Esper L, Gollob KJ, Teixeira MM, et al (2012) Current understanding of immunity to *Trypanosoma cruzi* infection and pathogenesis of Chagas disease. Seminars in Immunopathology 34: 753–770. 23076807 10.1007/s00281-012-0351-7 PMC3498515

[3] Huang H, Chan J, Wittner M, Jelicks LA, Morris SA, et al (1999) Expression of cardiac cytokines and inducible form of nitric oxide synthase (NOS2) in *Trypanosoma cruzi*-infected mice. J Mol Cell Cardiol 31: 75–88. 10072717 10.1006/jmcc.1998.0848

[4] Machado FS, Tyler KM, Brant F, Esper L, Teixeira MM, et al (2012) Pathogenesis of Chagas disease: time to move on. Front Biosci (Elite Ed) 4: 1743–1758. 22201990 10.2741/495 PMC3255071

[5] Machado FS, Souto JT, Rossi MA, Esper L, Tanowitz HB, et al (2008) Nitric oxide synthase-2 modulates chemokine production by *Trypanosoma cruzi*-infected cardiac myocytes. Microbes Infect 10: 1558–1566. 18951994 10.1016/j.micinf.2008.09.009 PMC2643379

[6] Coelho PS, Klein A, Talvani A, Coutinho SF, Takeuchi O, et al (2002) Glycosylphosphatidylinositol-anchored mucin-like glycoproteins isolated from *Trypanosoma cruzi* trypomastigotes induce in vivo leukocyte recruitment dependent on MCP-1 production by IFN-gamma-primed-macrophages. J Leukoc Biol 71: 837–844. 11994509

[7] Kayama H, Takeda K (2010) The innate immune response to *Trypanosoma cruzi* infection. Microbes Infect 12: 511–517. 20348008 10.1016/j.micinf.2010.03.005

[8] Monteiro AC, Schmitz V, Morrot A, de Arruda LB, Nagajyothi F, et al (2007) Bradykinin B2 Receptors of dendritic cells, acting as sensors of kinins proteolytically released by *Trypanosoma cruzi*, are critical for the development of protective type-1 responses. PLoS Pathog 3: e185. 18052532 10.1371/journal.ppat.0030185 PMC2098834

[9] Schmitz V, Svensjo E, Serra RR, Teixeira MM, Scharfstein J (2009) Proteolytic generation of kinins in tissues infected by *Trypanosoma cruzi* depends on CXC chemokine secretion by macrophages activated via Toll-like 2 receptors. J Leukoc Biol 85: 1005–1014. 19293401 10.1189/jlb.1108693

[10] Kanneganti TD (2010) Central roles of NLRs and inflammasomes in viral infection. Nat Rev Immunol 10: 688–698. 20847744 10.1038/nri2851 PMC3909537

[11] Garg NJ (2011) Inflammasomes in cardiovascular diseases. Am J Cardiovas Dis 1: 244–254. PMC3253520 22254202

[12] Ting JP, Lovering RC, Alnemri ES, Bertin J, Boss JM, et al (2008) The NLR gene family: a standard nomenclature. Immunity 28: 285–287. 18341998 10.1016/j.immuni.2008.02.005 PMC2630772

[13] van de Veerdonk FL, Netea MG, Dinarello CA, Joosten LA (2011) Inflammasome activation and IL-1beta and IL-18 proCessing during infection. Trends Immunol 32: 110–116. 21333600 10.1016/j.it.2011.01.003

[14] Foldes G, Liu A, Badiger R, Paul-Clark M, Moreno L, et al (2010) Innate immunity in human embryonic stem cells: comparison with adult human endothelial cells. PLoS One 5: e10501. 20463927 10.1371/journal.pone.0010501 PMC2864770

[15] Tousoulis D, Andreou I, Antoniades C, Tentolouris C, Stefanadis C (2008) Role of inflammation and oxidative stress in endothelial progenitor cell function and mobilization: therapeutic implications for cardiovascular diseases. Atherosclerosis 201: 236–247. 18599065 10.1016/j.atherosclerosis.2008.05.034

[16] Schultz K, Murthy V, Tatro JB, Beasley D (2007) Endogenous interleukin-1 alpha promotes a proliferative and proinflammatory phenotype in human vascular smooth muscle cells. Am J Physiol Heart Circ Physiol 292: H2927–2934. 17293495 10.1152/ajpheart.00700.2006

[17] Hirao K, Yumoto H, Takahashi K, Mukai K, Nakanishi T, et al (2009) Roles of TLR2, TLR4, NOD2, and NOD1 in pulp fibroblasts. J Dent Res 88: 762–767. 19734466 10.1177/0022034509341779

[18] Boyd JH, Mathur S, Wang Y, Bateman RM, Walley KR (2006) Toll-like receptor stimulation in cardiomyoCtes decreases contractility and initiates an NF-kappaB dependent inflammatory response. Cardiovasc Res 72: 384–393. 17054926 10.1016/j.cardiores.2006.09.011

[19] Dvinge H, Bertone P (2009) HTqPCR: high-throughput analysis and visualization of quantitative real-time PCR data in R. Bioinformatics. 25: 3325–3326. 10.1093/bioinformatics/btp578 PMC2788924 19808880

[20] Costales JA, Daily JP, Burleigh BA (2009) Cytokine-dependent and-independent gene expression changes and cell cycle bloCk revealed in *Trypanosoma cruzi*-infected host cells by comparative mRNA profiling. BMC Genomics 10: 252. 19480704 10.1186/1471-2164-10-252 PMC2709661

[21] Wen JJ, Garg NJ (2008) MitoChondrial generation of reactive oxygen species is enhanced at the Q(o) site of the complex III in the myoCardium of *Trypanosoma cruzi*-infected mice: beneficial effects of an antioxidant. J Bioenerg Biomembr 40: 587–598. 19009337 10.1007/s10863-008-9184-4 PMC6427913

[22] Dhiman M, Garg NJ (2011) NADPH oxidase inhibition ameliorates *Trypanosoma cruzi*-induced myoCarditis during Chagas disease. J Pathol 225: 583–596. 21952987 10.1002/path.2975 PMC4378678

[23] Liu W, Yin Y, Zhou Z, He M, Dai Y (2014) OxLDL-induced IL-1 beta secretion promoting foam cells formation was mainly via CD36 mediated ROS production leading to NLRP3 inflammasome activation. Inflamm Res 63: 33–43. 24121974 10.1007/s00011-013-0667-3

[24] Caetano BC, Carmo BB, Melo MB, Cerny A, dos Santos SL, et al (2011) Requirement of UNC93B1 reveals a critical role for TLR7 in host resistance to primary infection with *Trypanosoma cruzi* . J Immunol 187: 1903–1911. 21753151 10.4049/jimmunol.1003911 PMC3150366

[25] Bafica A, Santiago HC, Goldszmid R, Ropert C, Gazzinelli RT, et al (2006) Cutting edge: TLR9 and TLR2 signaling together account for MyD88-dependent control of parasitemia in *Trypanosoma cruzi* infection. J Immunol 177: 3515–3519. 16951309 10.4049/jimmunol.177.6.3515

[26] Silva GK, Gutierrez FR, Guedes PM, Horta CV, Cunha LD, et al (2010) Cutting edge: nucleotide-binding oligomerization domain 1-dependent responses account for murine resistance against *Trypanosoma cruzi* infection. J Immunol 184: 1148–1152. 20042586 10.4049/jimmunol.0902254

[27] Goncalves VM, Matteucci KC, Buzzo CL, Miollo BH, Ferrante D, et al (2013) NLRP3 controls *Trypanosoma cruzi* infection through a caspase-1-dependent IL-1R-independent NO production. PLoS Negl Trop Dis 7: e2469. 24098823 10.1371/journal.pntd.0002469 PMC3789781

[28] Silva GK, Costa RS, Silveira TN, Caetano BC, Horta CV, et al (2013) Apoptosis-assoCiated speck-like protein containing a caspase recruitment domain inflammasomes mediate IL-1beta response and host resistance to *Trypanosoma cruzi* infection. J Immunol 191: 3373–3383. 23966627 10.4049/jimmunol.1203293

[29] Oliveira AC, de Alencar BC, Tzelepis F, Klezewsky W, da Silva RN, et al (2010) Impaired innate immunity in Tlr4(-/-) mice but preserved CD8+ T cell responses against *Trypanosoma cruzi* in Tlr4-, Tlr2-, Tlr9- or Myd88-deficient mice. PLoS Pathog 6: e1000870. 20442858 10.1371/journal.ppat.1000870 PMC2861687

[30] Yin Y, Yan Y, Jiang X, Mai J, Chen NC, et al (2009) Inflammasomes are differentially expressed in cardiovascular and other tissues. Int J Immunopathol Pharmacol 22: 311–322. 19505385 10.1177/039463200902200208 PMC2847797

[31] Chawla A (2010) Control of macrophage activation and function by PPARs. Circ Res 106: 1559–1569. 20508200 10.1161/CIRCRESAHA.110.216523 PMC2897247

[32] Chawla A, Nguyen KD, Goh YP (2011) Macrophage-mediated inflammation in metabolic disease. Nat Rev Immunol 11: 738–749. 21984069 10.1038/nri3071 PMC3383854

[33] Bauernfeind F, Bartok E, Rieger A, Franchi L, Nunez G, et al (2011) Cutting Edge: Reactive Oxygen Species Inhibitors BloCk Priming, but Not Activation, of the NLRP3 Inflammasome. J Immunol 187: 613–617. 21677136 10.4049/jimmunol.1100613 PMC3131480

[34] Pan Q, Mathison J, Fearns C, Kravchenko VV, Da Silva Correia J, et al (2007) MDP-induced interleukin-1beta proCessing requires Nod2 and CIAS1/NALP3. J LeukoC Biol 82: 177–183. 17403772 10.1189/jlb.1006627

[35] Kanneganti TD, Lamkanfi M, Kim YG, Chen G, Park JH, et al (2007) Pannexin-1-mediated recognition of bacterial molecules activates the cryopyrin inflammasome independent of Toll-like receptor signaling. Immunity 26: 433–443. 17433728 10.1016/j.immuni.2007.03.008

[36] Hwang YS, Jeong M, Park JS, Kim MH, Lee DB, et al (2004) Interleukin-1beta stimulates IL-8 expression through MAP kinase and ROS signaling in human gastric carcinoma cells. Oncogene 23: 6603–6611. 15208668 10.1038/sj.onc.1207867

[37] Lo YY, Conquer JA, Grinstein S, Cruz TF (1998) Interleukin-1 beta induction of c-fos and collagenase expression in articular chondroCytes: involvement of reactive oxygen species. J Cell BioChem 69: 19–29. 9513043 10.1002/(sici)1097-4644(19980401)69:1<19::aid-jcb3>3.0.co;2-y

[38] van Bruggen R, Koker MY, Jansen M, van Houdt M, Roos D, et al (2010) Human NLRP3 inflammasome activation is Nox1-4 independent. Blood 115: 5398–5400. 20407038 10.1182/blood-2009-10-250803

[39] Sokolovska A, Becker CE, Ip WK, Rathinam VA, Brudner M, et al (2013) Activation of caspase-1 by the NLRP3 inflammasome regulates the NADPH oxidase NOX2 to control phagosome function. Nat Immunol 14: 543–553. 23644505 10.1038/ni.2595 PMC3708594

