# Supplementary material for: Caspase-1/ASC Inflammasome-Mediated Activation of IL-1β–ROS–NF-κB Pathway for Control of Trypanosoma cruzi Replication and Survival Is Dispensable in NLRP3−/− Macrophages
Source: PLoS One. 2014 Nov 5;9(11):e111539. doi: 10.1371/journal.pone.0111539 (PMC4221042; doi:10.1371/journal.pone.0111539)
Supplement: Table S1 — Oligonucleotides used in this study. (DOCX) [file pone.0111539.s001.docx]

**Table S1: Oligonucleotides used in this study**

**Human**

| **Gene name** | **Accession #** | **Forward primer (5’🡪 3’)** | **Reverse primer (5’🡪 3’)** | **Amplicon (bp)** |
| --- | --- | --- | --- | --- |
| IL-1β | NM_000576.2 | AACAGGCTGCTCTGGGATTCTCTT | ATTTCACTGGCGAGCTCAGGTACT | 92 |
| CCL5 | NM_002985.2 | TGCCTGTTTCTGCTTGCTCTTGTC | TGTGGTAGAATCTGGGCCCTTCAA | 92 |
| CXCL1 | NM_001511 | AAAGCTTGCCTCAATCCTGCATCC | TGCAGCTGTGTCTCTCTTTCCTCT | 168 |
| TNF-α | NM_000594 | TGGGCAGGTCTACTTTGGGATCAT | TTTGAGCCAGAAGAGGTTGAGGGT | 128 |
| GAPDH | NM_002046.5 | CCACTCCTCCACCTTTGAC | ACCCTGTTGCTGTAGCCA | 102 |
|  |  |  |  |  |

**Mouse**

| **Gene name** | **Accession #** | **Forward primer (5’🡪 3’)** | **Reverse primer (5’🡪 3’)** | **Amplicon (bp)** |
| --- | --- | --- | --- | --- |
| IL-1β | NM_008361.3 | GAG GAC ATG AGC ACC TTC TTT | GCC TGT AGT GCA GTT GTC TAA | 121 |
| CXCL1 | NM_008176.3 | GTGTCTAGTTGGTAGGGCATAAT | CAGTCCTTTGAACGTCTCTGT | 94 |
| TNF-α | NM_013693.3 | TTGCTCTGTGAAGGGAATGG | GGCTCTGAGGAGTAGACAATAAAG | 96 |
| GAPDH | NM_008084.3 | TGTGATGGGTGTGAACCACGAGAA | GAGCCCTTCCACAATGCCAAAGTT | 90 |
|  |  |  |  |  |

**T. cruzi**

| **Gene name** | **Accession #** | **Forward primer (5’🡪 3’)** | **Reverse primer (5’🡪 3’)** | **Amplicon (bp)** |
| --- | --- | --- | --- | --- |
| Tc18SrDNA | AF228685 | TAGTCATATGCTTGTTTC | GCAACAGCATTAATATACGC | 645 |
