# Supplementary material for: Caspase-1/ASC Inflammasome-Mediated Activation of IL-1β–ROS–NF-κB Pathway for Control of Trypanosoma cruzi Replication and Survival Is Dispensable in NLRP3−/− Macrophages
Source: PLoS One. 2014 Nov 5;9(11):e111539. doi: 10.1371/journal.pone.0111539 (PMC4221042; doi:10.1371/journal.pone.0111539)
Supplement: Table S2 — Inflammasome-related differential gene expression in THP-1 macrophages in response to LPS treatment (± ATP) in comparison to normal controls. (DOC) [file pone.0111539.s002.doc]

| **Table S2. Inflammasome-related differential gene expression in THP-1 macrophages in response to LPS treatment (± ATP) in comparison to normal controls** | | | | | | | | | | | |
| --- | --- | --- | --- | --- | --- | --- | --- | --- | --- | --- | --- |
| **LPS-treated vs control at 3 h** | | | | | **LPS-treated + ATP vs control at 3 h** | | | **LPS-treated + ATP vs LPS-treated at 3 h** | | | |
| **Gene name** | | **ddCt log ratio** | | **p value** | **Gene name** | **ddCt log ratio** | **p value** | **Gene name** | **ddCt log ratio** | **p value** | |
| CXCL1 | | -6.91 | | 0.000 | CXCL1 | -6.28 | 0.000 | FADD | -3.79 | | 0.013 |
| CXCL2 | | -7.06 | | 0.000 | CXCL2 | -6.75 | 0.000 | NLRC5 | -3.55 | | 0.014 |
| TNF | | -5.28 | | 0.000 | TNF | -4.97 | 0.000 | TXNIP | 1.80 | | 0.019 |
| RIPK2 | | -3.29 | | 0.000 | TXNIP | 5.03 | 0.000 |  |  | |  |
| NFKB1 | | -2.25 | | 0.000 | HSP90B1 | 2.24 | 0.000 |  |  | |  |
| NFKBIA | | -3.00 | | 0.000 | NFKBIA | -3.01 | 0.000 |  |  | |  |
| HSP90B1 | | 1.85 | | 0.000 | PYCARD | 2.87 | 0.000 |  |  | |  |
| PYCARD | | 2.71 | | 0.000 | RIPK2 | -3.04 | 0.000 |  |  | |  |
| MAPK1 | | 2.06 | | 0.000 | BIRC3 | -5.33 | 0.000 |  |  | |  |
| BIRC3 | | -4.92 | | 0.000 | NFKB1 | -2.12 | 0.000 |  |  | |  |
| RPL13A | | 2.22 | | 0.000 | RPL13A | 2.40 | 0.000 |  |  | |  |
| GAPDH | | 2.06 | | 0.000 | GAPDH | 2.24 | 0.000 |  |  | |  |
| FADD | | 6.71 | | 0.000 | PSTPIP1 | 2.29 | 0.000 |  |  | |  |
| PSTPIP1 | | 2.07 | | 0.000 | SUGT1 | 1.89 | 0.000 |  |  | |  |
| TXNIP | | 3.23 | | 0.000 | MAPK1 | 1.97 | 0.000 |  |  | |  |
| NLRC5 | | 5.92 | | 0.000 | HSP90AA1 | 2.26 | 0.000 |  |  | |  |
| CCL5 | | -2.61 | | 0.000 | CCL5 | -2.72 | 0.000 |  |  | |  |
| NLRP3 | | -1.47 | | 0.001 | B2M | 2.91 | 0.000 |  |  | |  |
| NFKBIB | | -1.77 | | 0.001 | ACTB | 1.59 | 0.003 |  |  | |  |
| CTSB | | 2.17 | | 0.002 | BIRC2 | -1.62 | 0.004 |  |  | |  |
| B2M | | 2.41 | | 0.002 | NLRP3 | -1.17 | 0.004 |  |  | |  |
| IRF2 | | 1.91 | | 0.003 | CTSB | 1.90 | 0.004 |  |  | |  |
| HSP90AA1 | | 1.56 | | 0.004 | PTGS2 | -4.01 | 0.011 |  |  | |  |
| SUGT1 | | 1.18 | | 0.005 | TAB1 | 1.76 | 0.015 |  |  | |  |
| PTGS2 | | -4.07 | | 0.010 | CCL2 | -3.51 | 0.019 |  |  | |  |
| NAIP | | 2.17 | | 0.011 | PEA15 | 0.93 | 0.033 |  |  | |  |
| TNFSF14 | | 2.52 | | 0.015 | TNFSF14 | 2.14 | 0.034 |  |  | |  |
| BIRC2 | | -1.29 | | 0.016 | CIITA | 1.41 | 0.038 |  |  | |  |
| CCL2 | | -3.37 | | 0.024 | NAIP | 1.70 | 0.039 |  |  | |  |
| ACTB | | 1.06 | | 0.034 | PANX1 | 0.94 | 0.045 |  |  | |  |
| TRAF6 | | 1.36 | | 0.048 | FADD | 2.92 | 0.048 |  |  | |  |
|  | |  | |  | IRF2 | 1.15 | 0.050 |  |  | |  |
|  | |  | |  | NFKBIB | -0.91 | 0.052 |  |  | |  |
|  | |  | |  |  |  |  |  |  | |  |
| **LPS-treated vs control at 18 h** | | | | | **LPS-treated+ATP vs control at 18 h** | | | **LPS-treated+ATP vs LPS-treated at 18 h** | | | |
| **Gene name** | | **ddCt log ratio** | | **p value** | **Gene name** | **ddCt log ratio** | **p value** | **Gene name** | **ddCt log ratio** | | **p value** |
| HSP90B1 | | 3.20 | | 0.000 | HSP90B1 | 3.81 | 0.000 | CHUK | 2.65 | | 0.014 |
| CXCL1 | | -4.62 | | 0.000 | CXCL2 | -4.52 | 0.000 | CXCL1 | 1.47 | | 0.010 |
| CXCL2 | | -4.76 | | 0.000 | TNF | -3.59 | 0.000 | CCL5 | 1.17 | | 0.059 |
| CCL5 | | -4.45 | | 0.000 | CASP1 | -2.36 | 0.000 | IRF1 | 1.63 | | 0.056 |
| TNF | | -3.23 | | 0.000 | CXCL1 | -3.15 | 0.000 | MAP3K7 | 0.67 | | 0.059 |
| CASP1 | | -2.30 | | 0.000 | CCL5 | -3.28 | 0.000 | NFKBIA | -1.16 | | 0.031 |
| IL1B | | -3.18 | | 0.001 | MAP3K7 | 1.61 | 0.000 | PEA15 | 0.84 | | 0.051 |
| CTSB | | 2.15 | | 0.002 | TAB2 | 2.09 | 0.000 | TAB2 | 0.96 | | 0.050 |
| CCL2 | | -4.78 | | 0.003 | CTSB | 2.45 | 0.001 |  |  | |  |
| MAPK12 | | 1.85 | | 0.004 | MAPK1 | 1.64 | 0.001 |  |  | |  |
| PTGS2 | | -4.58 | | 0.005 | CCL2 | -5.61 | 0.001 |  |  | |  |
| RPL13A | | 1.35 | | 0.006 | HSP90AA1 | 1.79 | 0.002 |  |  | |  |
| PYCARD | | 1.47 | | 0.007 | PTGS2 | -5.36 | 0.002 |  |  | |  |
| BIRC3 | | -2.72 | | 0.011 | CHUK | 3.22 | 0.004 |  |  | |  |
| HSP90AA1 | | 1.35 | | 0.011 | RPL13A | 1.39 | 0.005 |  |  | |  |
| MAPK1 | | 1.11 | | 0.011 | PEA15 | 1.29 | 0.005 |  |  | |  |
| MAP3K7 | | 0.93 | | 0.013 | NFKBIA | -1.57 | 0.006 |  |  | |  |
| RTC | | 1.73 | | 0.014 | PANX1 | 1.33 | 0.007 |  |  | |  |
| PSTPIP1 | | 1.13 | | 0.017 | TRAF6 | 1.82 | 0.012 |  |  | |  |
| MAPK3 | | 1.27 | | 0.022 | ACTB | 1.27 | 0.014 |  |  | |  |
| TAB2 | | 1.13 | | 0.025 | MAPK3 | 1.31 | 0.019 |  |  | |  |
| TRAF6 | | 1.53 | | 0.028 | CFLAR | -1.82 | 0.021 |  |  | |  |
| CFLAR | | -1.62 | | 0.037 | IKBKG | 1.47 | 0.024 |  |  | |  |
| RELA | | 0.80 | | 0.047 | IL1B | -1.95 | 0.029 |  |  | |  |
|  | |  | |  | B2M | 1.52 | 0.033 |  |  | |  |
|  | |  | |  | BIRC3 | -2.19 | 0.033 |  |  | |  |
|  | |  | |  | RELA | 0.87 | 0.032 |  |  | |  |
|  | |  | |  | TNFSF14 | 2.07 | 0.039 |  |  | |  |
|  | |  | |  | NAIP | 1.59 | 0.051 |  |  | |  |
|  | |  | |  | RTC | 1.29 | 0.055 |  |  | |  |
|  |  | |  | |  |  |  |  |  | |  |
| The 96-well RT^2^ Profiler^TM^ Human Inflammasome PCR Arrays (SA Biosciences/Qiagen) were probed in triplicate with cDNA from THP-1 macrophages treated with LPS for 3 h or 18 h (with or without ATP) as described in Materials and Methods. The Ct values from qPCR data were analyzed by using open source HTqPCR v.1.7 software package (v.2.13). All array data were normalized by Quantile method and filtered to exclude genes that exhibited Ct values >35. The relative expression level of each target gene in treated cells was calculated using the formula, fold change=2-∆Ct, where ∆Ct represents the Ct (sample) - Ct (control). LimmaCt in HT-qPCR package was employed for contrast analysis of all the groups included in experiment and identification of genes that were overall differentially expressed (p<0.05). | | | | | | | | | | | |
|
|
|
|
|
|
|
