# Supplementary material for: Caspase-1/ASC Inflammasome-Mediated Activation of IL-1β–ROS–NF-κB Pathway for Control of Trypanosoma cruzi Replication and Survival Is Dispensable in NLRP3−/− Macrophages
Source: PLoS One. 2014 Nov 5;9(11):e111539. doi: 10.1371/journal.pone.0111539 (PMC4221042; doi:10.1371/journal.pone.0111539)
Supplement: Table S3 — Inflammasome-related differential gene expression in non-phagoCytes at 24 h infection by Trypanosoma cruzi . (DOCX) [file pone.0111539.s003.docx]

| **Table S3. Inflammasome-related differential gene expression in non-phagocytes at 24 h infection by *Trypanosoma cruzi*** | | | | | | | |
| --- | --- | --- | --- | --- | --- | --- | --- |
| **Gene name** | **Fold change** | | | **Gene name** | **Fold change** | | |
|  | **HFF** | **HMVEC** | **HVSMC** |  | **HFF** | **HMVEC** | **HVSMC** |
| AIM2 | 2.6 | 1.1 | 1.51 | MAPK11 | -1.2 | -1.12 | 1.25 |
| BCL2 | 1.11 | -1.11 | 1.24 | MAPK12 | -1.94 | 1.19 | 1.42 |
| BCL2L1 | 1.55 | 1.25 | -1.2 | MAPK13 | -1.56 | -1.73 | 1.08 |
| BIRC2 | 1.7 | 1.09 | 1.41 | MAPK3 | -1.06 | 1.07 | 1.037 |
| BIRC3 | 1.563 | 3.609 | 1.13 | MAPK8 | -1.3 | -1.53 | -1.27 |
| CARD18 | 1.024 | -1 | 1.077 | MAPK9 | -1.46 | -1.51 | -1.42 |
| CARD6 | 2.38 | 1.66 | 1.14 | MEFV | 1.18 | -1.15 | -1.12 |
| CASP1 | 6.958 | 14.24 | 4.04 | MYD88 | 8.482 | 4.6 | 6.16 |
| CASP4 | 2.09 | 1.46 | 1.96 | NAIP | 1.5 | 1 | -1.18 |
| CASP5 | -1.011 | -1.05 | -1 | NFKB1 | 1.335 | 1.5 | 1.09 |
| CASP8 | 1.41 | 1.27 | 1.22 | NFKBIA | 2.692 | 2.21 | 1.29 |
| CCL2 | 3.395 | 1.31 | -1.09 | NFKBIB | 1.5 | 1.23 | 1.099 |
| CCL5 | 42.99 | 17.389 | 33.66 | NLRC4 | 1.18 | 1.28 | 1.17 |
| CCL7 | 1.41 | 1.04 | 1.06 | NLRC5 | 5.272 | 6.269 | 3.24 |
| CD40LG | 1.07 | -1.18 | 1.005 | NLRP1 | 1.16 | -1.32 | -1.16 |
| CFLAR | 2.77 | 1.78 | 1.68 | NLRP12 | 1.2 | 1.29 | 1.35 |
| CHUK | -1.27 | -1.46 | -1.31 | NLRP3 | 1.12 | 1.096 | 1.49 |
| CIITA | 1.09 | 1.32 | 1.78 | NLRP4 | 1.01 | 1.196 | 1.35 |
| CTSB | 1.09 | 1.26 | 1.11 | NLRP5 | 1.24 | 1.164 | 1.077 |
| CXCL1 | 2.578 | -1.63 | 1.05 | NLRP6 | -1.02 | 1.08 | 1.235 |
| CXCL2 | 1.69 | 1.32 | 1.24 | NLRP9 | 1.22 | 1.1 | 1.07 |
| FADD | 1.22 | -1.14 | 1.1 | NLRX1 | -1.38 | -1.26 | -1.12 |
| HSP90AA1 | 1.12 | 1.22 | 1.15 | NOD2 | 1.24 | 1.4 | -1 |
| HSP90AB1 | 1.026 | -1.17 | 1.03 | P2RX7 | 1.346 | 1.29 | 1.34 |
| HSP90B1 | 1.15 | -3.251 | -1.31 | PANX1 | 5.503 | 3.11 | 1.76 |
| IFNB1 | 87.572 | 18.65 | 6.21 | PEA15 | 1.18 | -1.53 | 1.04 |
| IFNG | 1.06 | -1.1 | 1.3 | PSTPIP1 | 1.054 | 1.2 | 1.06 |
| IKBKB | 1.28 | -1.17 | -1.09 | PTGS2 | 2.44 | 5.475 | 3.29 |
| IKBKG | 1.36 | 1.56 | 1.02 | PYCARD | 1.745 | 2.102 | 1.06 |
| IL12A | 2.07 | 1.38 | 2.06 | PYDC1 | 1.01 | -1.04 | 1.09 |
| IL12B | 1.01 | 1.05 | 1.11 | RAGE | -1.4 | -1.68 | 1.29 |
| IL18 | -1.35 | 1.007 | 1.08 | RELA | 1.72 | -1.06 | 1.17 |
| IL1B | 1.52 | 1.88 | 1.2 | RIPK2 | 1.68 | 2.33 | 1.76 |
| IL33 | -1.096 | -1.36 | 1.02 | SUGT1 | -1.68 | -1.38 | -1.35 |
| IL6 | 24.456 | 3.82 | 3.67 | TIRAP | 1.32 | -1.15 | -1.15 |
| IRAK1 | 1.18 | -1.15 | 1.14 | TNF | 1.14 | 1.25 | 1.07 |
| IRF1 | 5.197 | 3.536 | 2.1 | TNFSF11 | 1.04 | 1.05 | 1.1 |
| IRF2 | 2.99 | 1.8 | 1.58 | TNFSF14 | 1.22 | 1.69 | 1.74 |
| MAP3K7 | -2.609 | -1.48 | -1.72 | TNFSF4 | -1.2 | -10.162 | -1.33 |
| TAB1 | 1.32 | 1.6 | 1.31 | TRAF6 | 1.41 | 1.1 | 1.01 |
| TAB2 | 1.13 | -1.21 | -1.27 | TXNIP | -1.14 | 2.73 | 1.45 |
| MAPK1 | 1.57 | -2.389 | -1.63 | XIAP | 1.86 | 1.28 | 1.37 |

Infection of human foreskin fibroblasts (HFF), microvascular endothelial cells (HMVEC) and vascular smooth muscle cells (HVSMC), with *T. cruzi* for 24 h, followed by global gene expression profiling using the HG_U133 plus 2.0 Affymetrix chips is described by Costales et al [19]. The selected data-points for the differential expression of 84 genes included in the Inflammasome RT2 ProfilerTM PCR Array System (SA Biosciences) were filtered from Affymetrix datasets.
