# Supplementary material for: Caspase-1/ASC Inflammasome-Mediated Activation of IL-1β–ROS–NF-κB Pathway for Control of Trypanosoma cruzi Replication and Survival Is Dispensable in NLRP3−/− Macrophages
Source: PLoS One. 2014 Nov 5;9(11):e111539. doi: 10.1371/journal.pone.0111539 (PMC4221042; doi:10.1371/journal.pone.0111539)
Supplement: Table S4 — Ingenuity iReport analysis of inflammasome-related datasets in non-phagoCytes infected by T. cruzi . (DOCX) [file pone.0111539.s004.docx]

**Table S4. Ingenuity iReport analysis of inflammasome-related datasets in non-phagocytes infected by *T. cruzi***

| ***T. cruzi* infection of HFF vs Control (24 h)** | | | | | | | | |  |
| --- | --- | --- | --- | --- | --- | --- | --- | --- | --- |
|  | | Gene name | | # mols | | *P* value | |  |  |
| **Top biological and molecular functions** | | | | | | | |  |  |
| Cell death by apoptosis or necrosis (decreased) | | ↑AIM2, ↑BCL2L1, ↑BIRC2, ↑BIRC3, ↑CASP1, ↑CASP4, ↑CCL2,↑CCL5, ↑CFLAR, ↑CXCL1, ↑CXCL2, ↑IFNB1, ↑IL12A, ↑IL1B, ↑IL6, ↑IRF1, ↑IRF2, ↓MAP3K7, ↑MAPK1, ↓MAPK13,↑MYD88, ↑NAIP, ↑NFKB1A, ↑NFKB1B, ↑PTGS2, ↑RELA,↑RIPK2, ↑XIAP | | 28 | | 5.18E-18 | |  |  |
| Inflammatory response- Th1, NK and T cell activation, cell mediated response, dendritic cells/ macrophage migration | | ↑BCL2L1, BIRC2, ↑BIRC3, ↑CASP1, ↑CCL2,↑CCL5, ↑CXCL1, ↑CXCL2,↑IFNB1, ↑IL12A, ↑IL1B ,↑IL6,↑IRF1, ↑IRF2, ↓MAP3K7, ↑MYD88, ↑NFKB1A, ↑NFKB1B ↑PTGS2 ↑RELA,↑RIPK2 | | 22 | | 6.18E-10 | |  |  |
| **Canonical pathway** | | | | | | | | |  |
| PRR recognition of pathogens | | ↑CASP1, ↑CCL5, ↑IFNB1, ↑IL6, ↑IL12A, ↑IL1B, ↑MAPK1, MYD88, ↑RELA, ↑RIPK2 | | 10 | | 2.49E-15 | |  |  |
| IL17 signaling | | ↑CCL2,↑CCL5,↑IL6,↓MAP3K7,↑MAPK1, ↓MAPK13, ↑NFKB1A, ↑NFKB1B,↑PTGS2, ↑RELA | | 8 | | 2.54E-15 | |  |  |
| **Toxicity analysis** | |  | |  | |  | | |  |
| Gene regulation by PPARα | | ↑IL1B, ↓MAP3K7, ↑MAPK1, ↑NFKB1A, ↑NFKB1B ↑PTGS2 ↑RELA | | 7 | | 6.93E-10 | |  |  |

| ***T. cruzi* infection of HMVEC vs Control (24 h)** | | | | | |  |  |
| --- | --- | --- | --- | --- | --- | --- | --- |
|  | |  | # mols | | P value | | |
| **Top biological and molecular functions** | | | | | |  |  |
| Cell death – apoptosis/ necrosis decreased, bias for cell survival | | ↑BIRC3,↑CASP1, ,↑CCL5, ↑CFLAR, ↑CXCL1, ↑IFNB1, ↑IKBKG,↑IL1B,↑IL6, ↑IRF1, ↑IRF2, ↓MAPK1,↓MAPK9, ↓MAPK13,↑MYD88, ↑NFKB1, ↑NFKB1A,↓PEA15, ↑PTGS2, ↑RIPK2, ↑TNFS14, ↓TNFSF4, ↑TXNIP | 23 | 9.57E-21-1.09E-04 | |  |  |
| Inflammation- Th1, NK and T cell activation, dendritic cells/ macrophage migration | | ↑BIRC3, ↑CASP1, ↑CCL5, ↓CXCL1, ↑IFNB1, ↑IKBKG, ↑IL1B ,↑IL6, ↑IRF1, ↓MAPK9, ↑MYD88, ↑NFKB1, ↑NFKB1A, ↑PTGS2, ↑RIPK2, ↑TNFS14, ↓TNFSF4, ↑TXNI | 20 | 1.74E-13-9.99E-05 | |  |  |
| **Canonical pathway** | | | | | | |  |
| PRR recognition of pathogens | | ↑CASP1, ↑CCL5, ↑IFNB1, ↑IL6, ↑IL1B, ↓MAPK1, ↓MAPK9,↑MYD88, ↑NFKB1,↑RIPK2 | 10 | 4.83E-16 | |  |  |
| iNOS signaling | | ↑IKBKG, ↑IRF, ↓MAPK1,↑MYD88 , ↑NFKB1,↑NFKB1A,↑TAB1 | 8 | 5.37E-15 | |  |  |
| **Toxicity analysis** | | | | | | | |
| Gene regulation by PPARα | | ↑IKBG, ↑IL1B, ↓MAPK1, ↑NFKB1, ↑NFKB1A, ↑PTGS2, ↑TAB1 | 7 | 2.39E-10 | |  |  |

| ***T. cruzi* infection of HVSMC vs Control (24 h)** | | | | | |
| --- | --- | --- | --- | --- | --- |
|  | Gene name | # mols | | *P* value |  |
| **Top biological and molecular functions** | | | | | |
| Cell death (necrosis or apoptosis) | ↑AIM2, ↑CASP1, ↑CASP4, ↑CCL5, ↑CFLAR, ↑CIITA, ↑IFNB1, ↑IL12A, ↑IL6, ↑IRF1, ↑IRF2, ↓MAP3K7, ↓MAPK1, ↑MYD88, ↑PTGS2, ↑RIPK2, ↑TNFS14 | | 17 | 7.63E-17- 1.18E-03 |  |
| Cell proliferation of lymphocytes/T cells | ↑CCL5, ↑CFLAR, ↑CIITA, ↑IFNB1, ↑IL12A, ↑IL6, ↑IRF1, ↑IRF2, ↓MAP3K7, ↑MYD88, ↑PTGS2, ↑RIPK2, ↑TNFS14 | | 13 | 4.98E-13-1.09E-03 |  |
| Inflammatory activation of T and NK cells, phagocytes, movement of immune cells | ↑CASP1, ↑CCL5, ↑CIITA, ↑IFNB1, ↑IL12A, ↑IL6, ↑IRF2, ↓MAP3K7, ↑MYD88, ↑PTGS2, ↑TNFS14 | | 11 | 6.18E-10 |  |
| **Canonical pathway** | | | | | |
| PRR recognition of pathogens | ↑CASP1, ↑CCL5, ↑IFNB1, ↑IL6, IL12A, ↓MAPK1, ↑MYD88, ↑RIPK2 | | 8 | 1.34E-13 |  |

Infection of human foreskin fibroblasts (HFF), microvascular endothelial cells (HMVEC) and vascular smooth muscle cells (HVSMC) with *T. cruzi* for 24 h, followed by global gene expression profiling using the HG_U133 plus 2.0 Affymetrix chips is described by Costales et al {}. The selected data-points for the differential expression of 84 genes included in the Inflammasome RT2 ProfilerTM PCR Array System (SA Biosciences) were filtered from Affymetrix datasets, and submitted to Ingenuity iReport for biological and statistical analysis of the experimental data.
